# Pattern formation by turbulent cascades

**DOI:** 10.1038/s41586-024-07074-z

**Published:** 2024-03-20

**Authors:** Xander M. de Wit, Michel Fruchart, Tali Khain, Federico Toschi, Vincenzo Vitelli

**Affiliations:** 1 Department of Applied Physics and Science Education, Eindhoven University of Technology, Eindhoven, The Netherlands; 2 Gulliver, ESPCI Paris, Université PSL, CNRS, Paris, France; 3 James Franck Institute, The University of Chicago, Chicago, IL USA; 4 CNR-IAC, Rome, Italy; 5 Kadanoff Center for Theoretical Physics, The University of Chicago, Chicago, IL USA

**Keywords:** Physics, Fluid dynamics, Statistical physics, thermodynamics and nonlinear dynamics

## Abstract

Fully developed turbulence is a universal and scale-invariant chaotic state characterized by an energy cascade from large to small scales at which the cascade is eventually arrested by dissipation^1–6^. Here we show how to harness these seemingly structureless turbulent cascades to generate patterns. Pattern formation entails a process of wavelength selection, which can usually be traced to the linear instability of a homogeneous state^7^. By contrast, the mechanism we propose here is fully nonlinear. It is triggered by the non-dissipative arrest of turbulent cascades: energy piles up at an intermediate scale, which is neither the system size nor the smallest scales at which energy is usually dissipated. Using a combination of theory and large-scale simulations, we show that the tunable wavelength of these cascade-induced patterns can be set by a non-dissipative transport coefficient called odd viscosity, ubiquitous in chiral fluids ranging from bioactive to quantum systems^8–12^. Odd viscosity, which acts as a scale-dependent Coriolis-like force, leads to a two-dimensionalization of the flow at small scales, in contrast with rotating fluids in which a two-dimensionalization occurs at large scales^4^. Apart from odd viscosity fluids, we discuss how cascade-induced patterns can arise in natural systems, including atmospheric flows^13–19^, stellar plasma such as the solar wind^20–22^, or the pulverization and coagulation of objects or droplets in which mass rather than energy cascades^23–25^.

## Abstract (web summary)

Turbulent energy cascades can be arrested by non-dissipative viscosities, resulting in pattern formation at intermediate length scales.

## Main

**Fig. 1:**
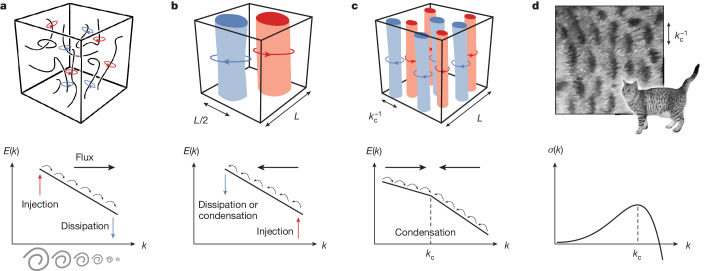
Cascade-induced pattern formation. **a**, Direct energy cascade: in a turbulent 3D fluid, energy injected at large scales (red arrow) is transferred to smaller and smaller length scales (black arrows) to microscopic length scales in which dissipation occurs (blue arrow), as captured by the so-called energy spectrum *E*(*k*), which describes how much kinetic energy is contained in modes with wavenumber *k*. The energy transfer across scales can be traced to vortices breaking up into smaller and smaller vortices up to dissipative scales. This mechanism is intrinsically nonlinear: it relies on triadic couplings between the modes of the system. **b**, Inverse energy cascade: in a turbulent 2D fluid, or in a rotating 3D fluid, there is instead a transfer of energy from the scale in which energy is injected (red arrow) to larger and larger scales, and the energy is either dissipated or piles up at the largest scale available (blue arrow), the size of the system. Correspondingly, vortices merge together until only a single positive vortex and a single negative vortex remain, both of which have approximately half the size *L* of the system. Inverse cascades can also arise in 3D from mirror symmetry breaking^4,55,56^ or by imposing large-scale shear^57^. **c**, In a hypothetical situation in which a direct cascade and an inverse cascade can be put together in the right order (black arrows in the figure), energy will be transferred to an intermediate length scale ${k}_{{\rm{c}}}^{-1}$, leading to the appearance of structures with a characteristic size ${k}_{{\rm{c}}}^{-1}$ independent of the size *L* of the system. This nonlinear wavelength selection mechanism relying on combined turbulent cascades can be seen as an instance of pattern formation. **d**, Standard pattern formation from a linear instability: the wavelength ${k}_{{\rm{c}}}^{-1}$ corresponding to the most unstable linear mode (that is, the one with the largest growth rate *σ*(*k*)) is selected. As an example, we have shown the coat pattern of a cat.

Fully developed turbulence is a highly chaotic non-equilibrium state in which energy is transferred across scales through a nonlinear mechanism known as a turbulent cascade^1–6^. Although cascades occur in diverse contexts ranging from optical fibres to solid plates^26–29^, their most iconic manifestation is in fluids. Heuristically, large eddies, typically created by the injection of energy at macroscopic scales, break up into smaller and smaller eddies. This energy transfer towards small scales, called a direct or forward cascade, is eventually arrested by dissipation (Fig. 1a). Away from the scales at which energy is injected and dissipated, turbulence is universal and scale invariant.

We start with the almost paradoxical question of whether turbulence can be harnessed to generate patterns. Our approach to tackle this task rests on the simple observation that different classes of turbulent cascades exist^4^. For example, turbulence in two-dimensional (2D) and rotating fluids has a tendency to transfer energy towards larger scales in what is known as an inverse cascade (Fig. 1b). Here we consider what happens when a direct cascade is combined with an inverse cascade as shown in Fig. 1c. Energy is transferred to an intermediate length scale ${k}_{{\rm{c}}}^{-1}$ (*k* are wavenumbers, so their inverses are lengths) both from smaller and larger scales, depending on where energy is injected. As energy accumulates around that scale, structures emerge with characteristic size ${k}_{{\rm{c}}}^{-1}$, which is neither the size of the system nor the smallest scales at which dissipation typically occurs. This spectral condensation at intermediate scales requires the mechanism responsible for arresting both cascades to be non-dissipative. As we shall see, nature has found an elegant solution to this problem: a viscosity that does not dissipate energy^9,12^, variously known as odd viscosity^8^, Hall viscosity^10^ or gyroviscosity^11^.

Before exploring potential realizations, let us compare and contrast this scenario with the textbook picture of pattern formation represented in Fig. 1d. In its simplest form, pattern formation originates from the linear instability of a homogeneous system: the length scale ${k}_{{\rm{c}}}^{-1}$, corresponding to the maximum of the growth rate *σ*(*k*), is selected because the corresponding mode grows faster, and sets the characteristic size of the emerging pattern. Although nonlinearities are important in saturating the growth and selecting the precise shape of the pattern, they play only a part once the linear instability has set in. This linear mechanism is at play in many areas of science^7,12^. By contrast, in the mechanism shown in Fig. 1c, it is the nonlinear interaction between modes that gives rise to the turbulent cascade.

**Fig. 2:**
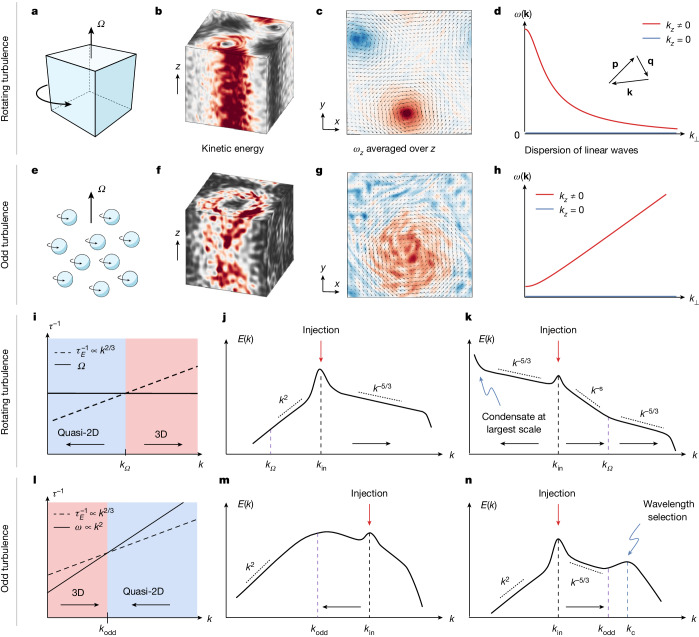
Rotating versus odd turbulence. **a**–**n**, We compare turbulence in a fluid rotating with high frequency **Ω** (**a**–**d** and **i**–**k**) and a fluid with high odd viscosity (**e**–**h** and **l**–**n**). **a**–**h**, Both fluids are characterized by a rotation direction **Ω** (along *z*), making them anisotropic and chiral. The rotation is global in rotating fluids (**a**). It is induced at microscopic scales in odd fluids, for instance, by particles that all spin in the same direction **Ω** (**e**). In both cases, the flow becomes 2D, with column-like structures aligned with **Ω**, as seen in the kinetic energy (**b**,**f**) and the *z*-averaged vertical vorticity ${\langle {\omega }_{z}\rangle }_{z}$ (**c**,**g**) obtained from simulations. The two-dimensionalization originates from the decorrelation by waves in the fluid (inertial waves in **d** and odd waves in **h**) of the triads by which energy transfer occurs (**d**, inset). Modes with *k*_*z*_ ≠ 0 have finite frequencies (red lines) and quickly decorrelate, whereas modes with no vertical variation (*k*_*z*_ = 0, blue lines) all have *ω* = 0. **i**–**n**, To predict the direction of the cascades (black arrows), we compare the inverse frequency of waves with the time over which energy transfer takes place (the eddy turnover time ${\tau }_{E}^{-1}\propto {k}^{2/3}$). In rotating fluids (**i**), the flow is quasi-2D at small wavenumbers (blue region) and isotropic (3D) at large wavenumbers (once ${\tau }_{E}^{-1} > \Omega$, red region). In odd fluids (**l**), we expect the flow to be quasi-2D at large wavenumbers (blue region) and isotropic at low *k* (once ${\tau }_{E}^{-1} > {\tau }_{{\rm{o}}{\rm{d}}{\rm{d}}}^{-1}$, red region). The crossover point defines a characteristic scale *k*_odd_, in analogy with the Zeman scale *k*_*Ω*_ in rotating fluids. We sketch cascades in the energy spectra when the injection scale is smaller (**j**,**m**) and larger (**k**,**n**) than the characteristic scale. In rotating fluids, there is a direct cascade of energy above the rotation (Zeman) scale (**j**) and an inverse cascade below (**k**). This situation is known as a split cascade^4^. In odd fluids, we expect the situation to be reversed: energy cascades directly for wavenumbers below *k*_odd_ (**n**) and inversely above (**m**), causing a pile-up of energy at the odd viscosity length scale and arresting both cascades. The pile-up is saturated by viscous dissipation, leading to a bump in the energy spectrum at another scale *k*_c_.

To realize the mixed cascade of Fig. 1c, we first need to turn a direct cascade into an inverse cascade. This can be achieved by simply rotating the fluid at high velocities^2,4^, as shown in Fig. 2. The Coriolis force **f**_**Ω**_ = 2*ρ***v** × **Ω** (where **v**(*t*, **x**) is the velocity field, *ρ* is the density, **Ω** is the rotation vector and × is the vector product) tends to align vortex lines with the rotation axis, without injecting or dissipating energy. As the rotation speed increases, the vortex tangle becomes more and more polarized, which induces a two-dimensionalization of the flow. This prevents vortex stretching and leads to an inverse energy cascade similar to the case of 2D fluids. Eventually, the energy condenses into two vortices of opposite vorticity. As the inverse cascade proceeds all the way to the largest scales, this condensation occurs only at the size of the system (Figs. 1b and 2b,c).

## Turbulence with odd viscosity

For our purposes, we need an inverse cascade at small scales (large wavevectors) only (Fig. 1c). This could be produced by a scale-dependent version of the Coriolis force that would involve gradients of the velocity, in a way similar to a viscosity term, so that it is negligible at large scales. To do so, we consider a situation in which rotation is induced at microscopic scales, for instance by spinning particles large enough to be inertial (Fig. 2e). It turns out that such a system has an antisymmetric part in its viscosity tensor *η*_*ijkℓ*_ ≠ *η*_*kℓij*_, known as odd viscosity. Like the Coriolis force, the antisymmetric, or odd, part of the viscosity tensor does not contribute to energy dissipation or injection as it drops out from the energy balance equation^30^. Odd viscosities arise in various experimental systems breaking time-reversal and inversion symmetry at the microscopic scale^8,9^, including magnetized polyatomic gases^31^, magnetized graphene^10^ and active colloids^32^.

To mathematically account for the effect of odd viscosity, we consider a simple extension of the Navier–Stokes equations

1 $$\begin{array}{r}{D}_{t}{\bf{v}}=-{\boldsymbol{\nabla }}P+\nu \Delta {\bf{v}}+{\nu }_{{\rm{odd}}}{{\bf{e}}}_{z}\times \Delta {\bf{v}}+{\bf{f}}(t,{\bf{x}})\end{array}$$

with the incompressibility condition **∇** ⋅ **v** = 0. Here, *D*_*t*_ = ∂_*t*_ + **v** ⋅ **∇** is the convective derivative and **f** is an external forcing representing energy injection, *P* is the pressure, *ν* = *η*/*ρ* is the familiar shear viscosity, *ν*_odd_ = *η*_odd_/*ρ* is a particular combination of odd viscosities (see Supplementary Information for the general case) and **e**_*z*_ is the unit vector along *z* (the direction set by the magnetic field or rotation axis). Equation (1) can be seen as a nonlinear diffusion equation for momentum with an antisymmetric cross-diffusion coefficient *ν*_odd_. The resulting odd viscosity term *ν*_odd_**e**_*z*_ × Δ**v** (or −*ν*_odd_*k*^2^**e**_*z*_ × **v**(**k**) in wavenumber space) can be seen as a scale-dependent Coriolis force. Both are non-dissipative and anisotropic (Methods). The additional Laplacian ensures that the action of *ν*_odd_ vanishes for small wavenumbers, as needed to arrest the turbulent cascade at intermediate scales.

## Two-dimensionalization  by odd waves

Direct numerical simulations of the Navier–Stokes equations (Methods) in Fig. 2 confirm that strong odd viscosity fluids can exhibit features similar to quickly rotating fluids such as Taylor columns and quasi-two-dimensionalization^2^ (compare Fig. 2a–c with Fig. 2e–g). The two-dimensionalization of the flow can be heuristically justified using a generalization of the Taylor–Proudman argument to odd fluids, where the convective term is neglected, and which yields ∂_*z*_Δ**v** = **0** (Supplementary Information).

To account for the role of the convective term, we now turn to the analysis of the nonlinear energy transfer, which governs the redistribution of energy among scales^2,4^. The distribution of energy among scales is described by the energy spectrum $E(k,t)=\frac{1}{2}{\left\langle \parallel {\bf{v}}(t,{\bf{k}}){\parallel }^{2}\right\rangle }_{k\le \parallel {\bf{k}}\parallel  < k+1}$ averaged over a spherical shell. Its evolution is captured by the energy balance equation ∂_*t*_*E* = − *T* − *νk*^2^*E* + *F*, in which *F* represents the forcing and *T* the nonlinear energy transfer between scales.

As odd viscosity is non-dissipative, it does not act as an energy source or sink. However, it has an indirect effect on the energy transfer, because it induces waves in the fluid, that oscillate at a frequency *ω*(**k**) = ±*ν*_odd_*k*_*z*_∣*k*∣ (Fig. 2h and Supplementary Information). The transfer described by *T* arises through interactions between three modes with wavenumbers **k**, **p** and **q** that satisfy **k** + **p** + **q** = **0** (called a triad; Fig. 2d, inset). Because of the odd waves described above, the different modes in a triad quickly go out of phase with each other. This suppresses the nonlinear energy transfer, except for modes with *k*_*z*_ = 0, which all have *ω* = 0 (Fig. 2h, blue line) and therefore do not decorrelate. These 2D modes form a so-called slow (or resonant) manifold that contributes to most of the nonlinear energy transfer, giving rise to an inverse cascade. This can be seen from the expression of the energy transfer *T* ∝ e^i[*ω*(**k**)+*ω*(**p**)+*ω*(**q**)] *t*^ (see the Methods for details; recall that the time average of e^i*ωt*^ vanishes when *ω* ≠ 0).

## Scaling theory of the arrested cascade

In a turbulent flow, the lifespan of a typical eddy is called the turnover time *τ*_*E*_, and its inverse is called the eddy turnover frequency. The processes transferring energy across scales occur over a few turnover times. To assess whether odd waves suppress the energy transfer, we compare the eddy turnover frequency ${\tau }_{{\rm{E}}}^{-1}$ with the frequency *ω*(**k**) of odd waves. Assuming *k*_*z*_ ≈ *k* (motivated by the isotropization at small *k*), we look for the scale *k*_odd_ such that $\omega (k={k}_{{\rm{odd}}})={\tau }_{{\rm{E}}}^{-1}\,(k={k}_{{\rm{odd}}})$ (Fig. 2l). We estimate the eddy turnover frequency ${\tau }_{{\rm{E}}}^{-1}=k{v}_{k}\propto {k}^{2/3}{{\epsilon }}^{1/3}$ from the rate of dissipation of energy at small scales *ϵ* using the Kolmogorov scaling valid at *k* ≪ *k*_odd_, and find

2 $${k}_{{\rm{odd}}}\equiv {{\epsilon }}^{1/4}{\nu }_{{\rm{odd}}}^{-3/4}.$$

When *k* ≫ *k*_odd_, the effect of odd viscosity is important: the contribution of 3D triads to the energy transfer averages to zero over the lifespan of a typical eddy, and we expect quasi-2D behaviour. By contrast, when *k* ≪ *k*_odd_, the effect of odd viscosity is negligible and we expect normal 3D behaviour. This is summarized in Fig. 2l. As a consequence, both a direct and an inverse cascade are arrested when they approach the odd viscosity wavenumber *k*_odd_, because of the inherent tendency to cascade in the opposite direction beyond that wavenumber (Fig. 2m,n). The direct cascade dominates when energy is injected below *k*_odd_ (Fig. 2n), whereas the inverse cascade dominates when energy is injected above *k*_odd_ (Fig. 2m).

Figure 2 compares the cases of odd and rotating fluids. In the case of rotating turbulence^4,33–36^, odd waves are replaced by so-called inertial waves with dispersion *ω*_**Ω**_(**k**) = ±2**Ω** × **k**/*k* (Fig. 2d), and the scale *k*_odd_ is replaced by the so-called Zeman scale *k*_*Ω*_ = *Ω*^3/2^*ϵ*^−1/2^ (refs. ^37,38^). Comparing Fig. 2i with Fig. 2l shows that, crucially, the order of the 3D direct cascade and the quasi-2D inverse cascade are permuted in rotating and odd fluids. As a consequence, the fluxes are convergent in the case of odd turbulence, whereas they are divergent in the case of rotating turbulence, and the pattern formation effect is thus observed only in the former scenario.

## Wavelength selection in the energy spectrum

We now refine the intuitive picture in Fig. 1c and show that two length scales, rather than a single one, are implicated in cascade-induced pattern formation. To do so, we develop a scaling theory based on dimensional analysis^33,37,39–42^, focusing on the case in which energy is injected at large-scale *k*_in_ < *k*_odd_ and the direct cascade dominates.

As the cascade is generated by nonlinear triadic interactions, we expect that it is related to the corresponding correlation time *τ*_3_(*k*). Assuming energy conservation and locality in the scale of the cascade, dimensional analysis leads to $E(k)=C\,{[{\epsilon }/{\tau }_{3}(k)]}^{1/2}\,{k}^{-2}$ in which *C* is a constant^39,40,42^.

In the absence of odd viscosity, or when it is negligible (*k* ≪ *k*_odd_), the only time scale available is the eddy turnover time ${\tau }_{{\rm{E}}}(k)={[k{v}_{k}]}^{-1}\,=$ ${k}^{-3/2}{E}^{-1/2}(k)$, leading to the Kolmogorov spectrum

3 $$E(k)\propto {{\epsilon }}^{2/3}{k}^{-5/3}\qquad (k\ll {k}_{{\rm{odd}}}).$$

When odd viscosity is dominant (*k* ≫ *k*_odd_), the relevant time scale is given by the frequency of odd waves *ω*(*k*) = *ν*_odd_*k*^2^ (again, we assume *k*_*z*_ ≈ *k*), leading to

4 $$E(k)\propto {{\epsilon }}^{1/2}{\nu }_{{\rm{odd}}}^{1/2}\,{k}^{-1}\qquad (k\gg {k}_{{\rm{odd}}}).$$

As a point of comparison, the relevant time scale is *Ω*^−1^ in rotating turbulence, so this argument leads to a different scaling *E* ∝ *k*^−2^ (refs. ^33,40^).

The preceding argument shows that the cascade starts to get arrested when it reaches *k*_odd_, leading to an amplification of the modes with wavenumbers *k* > *k*_odd_. The relative amplification due to odd viscosity can be described by the ratio between the modified spectrum *E*(*k*) given by equation (4) and the Kolmogorov spectrum *E*_0_(*k*) given by equation (3) that would occur in the absence of odd viscosity. Ignoring first the effect of dissipation, this yields *E*/*E*_0_ = 1 for *k* ≪ *k*_odd_ and $E/{E}_{0}\propto {(k/{k}_{{\rm{odd}}})}^{2/3}$ for *k* ≫ *k*_odd_. As energy piles up at wavevectors larger than *k*_odd_, it is eventually saturated by viscous dissipation, leading to a maximum in *E*/*E*_0_ after which the spectrum decays dissipatively.

By balancing energy injection and viscous dissipation, we can find the position *k*_c_ of the maximum as (see Methods)

5 $${k}_{{\rm{c}}}\propto {{\epsilon }}^{1/4}{\nu }^{-1/2}{\nu }_{{\rm{odd}}}^{-1/4}.$$

The magnitude of the spectral condensation can be estimated as the height of the peak $E({k}_{{\rm{c}}})/{E}_{0}({k}_{{\rm{c}}})\propto {({\nu }_{{\rm{odd}}}/\nu )}^{1/3}$. The ratio *ν*_odd_/*ν* thus controls the height of the peak. According to kinetic theory calculations corroborated by experimental measurements, this ratio increases linearly with the time-reversal breaking field (for example, the spinning speed in Fig. 2e or the applied magnetic field; see Methods).

The overall picture, summarized in Fig. 2n, involves the two length scales *k*_odd_ and *k*_c_ defined in equations (2) and (5). As the direct cascade (black arrow) approaches *k*_odd_ (purple dashed line), it is gradually arrested: the rate of energy transfer from scale to scale decreases as *k* increases. This leads to the condensation of kinetic energy in wavenumbers *k* > *k*_odd_. In turn, the amplification of these modes leads to an increase in viscous dissipation, and the energy spectrum exhibits a maximum deviation from the Kolmogorov spectrum at a characteristic wavenumber *k*_c_ (blue dashed line).

## Simulations of the odd Navier–Stokes equations

**Fig. 3:**
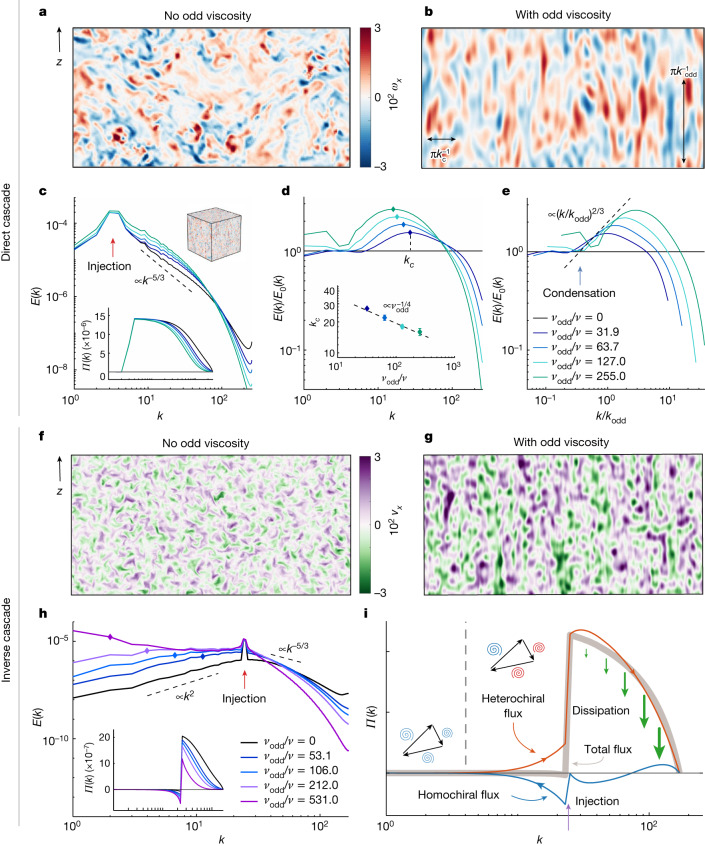
Odd waves induce wavelength selection and flux loops. **a**–**i**, We perform direct simulations of the Navier–Stokes equation without and with odd viscosity. In **a**–**e**, energy is injected at wavenumbers *k*_in_ < *k*_odd_ and the direct cascade dominates. In **f**–**i**, *k*_in_ > *k*_odd_ and the inverse cascade dominates. **a**,**b**, Slices of the in-plane component *ω*_*x*_ of the vorticity with *k*_in_ < *k*_odd_. Without odd viscosity (**a**), vortices of all sizes are present. With odd viscosity (**b**, in which *ν*_odd_/*ν* = 255), characteristic horizontal and vertical scales ${k}_{{\rm{c}}}^{-1}$ and ${k}_{{\rm{odd}}}^{-1}$ emerge (black arrows). This wavelength selection originates from the arrest of the direct cascade near *k*_odd_. **c**, Energy spectrum *E*(*k*) and flux *Π*(*k*) (inset) obtained from simulations, for different values of odd viscosity (legend in **e**). Energy flows from the injection scale *k*_in_ (red arrow) towards larger *k*, as evidenced by the positive energy flux *Π*(*k*). The cascade is progressively arrested near *k*_odd_ and energy piles up, triggering viscous dissipation. **d**, The relative energetic amplification and/or attenuation due to odd viscosity is measured by the compensated spectrum *E*(*k*)/*E*_0_(*k*) (where *E*_0_(*k*) is the energy spectrum without odd viscosity), which peaks at a scale *k*_c_ (diamonds). The peak position *k*_c_ decreases as odd viscosity increases (inset), as predicted by scaling arguments (dashed line; see equation (5)). **e**, Plotting the compensated spectra against *k*/*k*_odd_ confirms that condensation begins near *k*_odd_ (blue arrow) and follows the scaling prediction (dashed line; see equations (2)–(4)). **f**,**g**, Slices of the in-plane velocity component *v*_*x*_ when *k*_in_ > *k*_odd_. We visualize *v*_*x*_ instead of *ω*_*x*_ to emphasize the large scales. Without odd viscosity (**f**), structures of all scales are present, dominated by the injection scale. With odd viscosity (**g**, in which *ν*_odd_/*ν* = 212), secondary features with larger sizes appear because of the arrest of the inverse cascade. **h**, Energy spectrum *E*(*k*) and flux *Π*(*k*) obtained from the simulations (diamonds indicate *k*_odd_). **i**, The inverse cascade is arrested by a flux-loop mechanism, as evidenced by a decomposition of the flux in homochiral (blue) and heterochiral (red) channels that correspond, respectively, to triads with different or same signs of helicity. In **i**, we have used hyperdissipation in the simulations to highlight the flux loop (Extended Data Fig. 1a iv).

To put this scenario to test, we numerically integrate the Navier–Stokes equation (1) using a parallelized pseudo-spectral solver (Methods). In a normal fluid, eddies of all sizes can be found in the statistical steady state (Fig. 3a). In the presence of odd viscosity, the turbulent state selects a dominant scale, as shown in the visualizations of the vorticity field in Fig. 3b. The features manifest as vertically aligned, intermediate scale structures, as expected from the quasi-2D nature of the system. A direct cascade occurs when energy is injected at large scales (*k*_in_ < *k*_odd_). As predicted, we find that this turbulent cascade is arrested because of odd viscosity. This can be seen from the net flux of energy $\varPi (k)=\sum _{{k}^{{\prime} } < k}T({k}^{{\prime} })$, which gradually decays as *k* passes *k*_odd_ (Fig. 3c, inset).

This gradual arrest of the cascade near *k*_odd_ leads to spectral condensation at intermediate scales. Quantitatively, the spectral condensation and wavelength selection can be better appreciated from the relative energetic amplification of each mode *E*(*k*)/*E*_0_(*k*) shown in Fig. 3d. Rescaling the wavenumbers by *k*_odd_ (Fig. 3e), we observe an approximate collapse of the curves compatible with the scaling predicted in the previous paragraph. The condensation peaks around a wavenumber *k*_c_, which we can compare quantitatively with our scaling prediction equation (5) (Fig. 3d, inset). An extension of our scaling theory taking into account the anisotropy of the flow (Methods) reveals the visual meaning of the two length scales involved in cascade-induced patterns: ${k}_{c}^{-1}$ manifests predominantly in the horizontal direction, whereas the typical vertical scale is mainly given by ${k}_{{\rm{odd}}}^{-1}$ (Fig. 3b, black arrows).

## Flux loops and helicity conservation

When energy is injected at *k*_in_ > *k*_odd_ (Fig. 3f–i), we expect an inverse cascade to be arrested by odd viscosity. This is the case, as evidenced by snapshots of the steady state, that exhibit scales larger than the injection scale (Fig. 3g). In contrast with the case of the arrested direct cascade, here energy gets piled up at large scales, in which viscous dissipation is not an effective saturation mechanism. Instead, what prevents energy blow-up is a mechanism known as flux-loop cascade^4^: energy goes from the small injection scale to large scales and then back to even smaller scales where it is dissipated. To see that, we decompose the energy flux into heterochiral (red) and homochiral (blue) channels, that correspond, respectively, to triads with different or same signs of helicity. Helicity is the volume integral of **v** ⋅ **ω**, where **ω** = **∇** × **v** is the vorticity, and it is an invariant of the inviscid Navier–Stokes equation. The conservation of helicity is not affected by odd viscosity (Methods). As shown in Fig. 3i, the heterochiral flux (red) tends to cascade directly, whereas the homochiral flux (blue) tends to cascade inversely. Below the injection scale, both fluxes cancel exactly, leading to a vanishing net flux (grey line). In the case of the inverse cascade, the resulting pattern is less visible than in the direct cascade, because the energy is deposited over a more broadband range *k*_odd_ < *k* < *k*_in_.

## Pattern-induced cascades beyond odd fluids

**Fig. 4:**
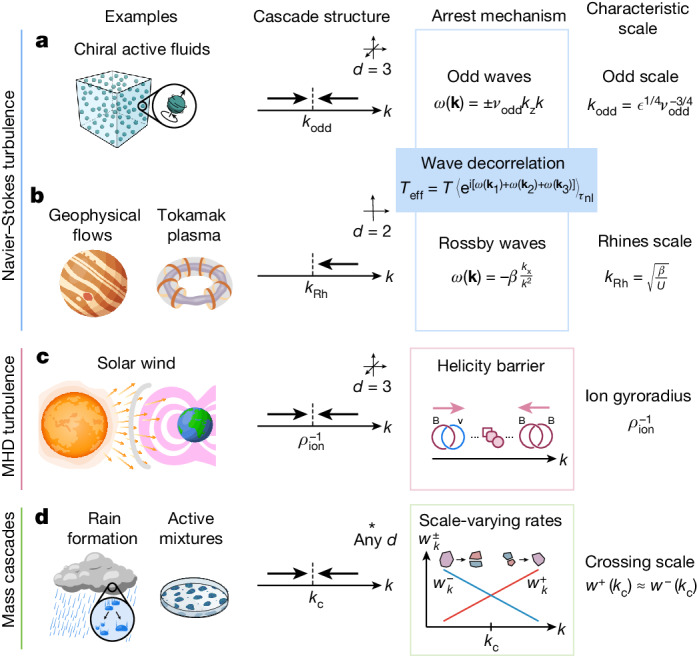
Cascade-induced pattern formation across domains. **a**–**d**, Cascade-induced scale selection can occur in systems ranging from Navier–Stokes turbulence (**a**,**b**) and magnetohydrodynamics (MHD) turbulence (**c**) to mass cascades (**d**). **a**, Chiral active fluids are an example of fluids with odd viscosity. As demonstrated in Fig. 3, these fluids are expected to exhibit a double arrested cascade at high enough *ν*_odd_/*ν* and Reynolds number (see the Methods for a discussion on orders of magnitude). We have interpreted this phenomenon as the result of a decorrelation of wavenumber triads by odd waves. **b**, In 2D geophysical flows and plasma, an arrested inverse cascade associated with wavelength selection occurs^13–19^. It can be seen as the consequence of the decorrelation of triads by Rossby (or drift) waves, which set the characteristic scale *k*_Rh_ known as the Rhines scale. **c**, A double arrested cascade has been predicted in the solar wind, based on the properties of inviscid invariants of finite Larmor radius MHD^20–22^. This mechanism, known as a helicity barrier, relies on the change of nature of an inviscid invariant, which interpolates between cross-helicity and magnetic helicity (these quantities cascade in opposite directions). **d**, Scale selection can also occur in mass cascades, ranging from the stationary distribution of raindrop sizes that would occur in steady-state conditions^24^ to smoke aerosols^25^. This arises from the balance between coalescence and breakup of the droplets, which effectively have scale-varying rates (${w}_{k}^{\pm }$, red and blue curves in the schematic). Similar phenomenology arises in active mixtures^46–51^, although not necessarily with a flux across scales. In the Methods, we provide a minimal model of mass cascade exhibiting scale selection.

Our analysis demonstrates that the non-dissipative arrest of turbulent cascades provides a mechanism of wavelength selection. The decorrelation of triads by waves and the subsequent emergence of a resonant manifold are not unique to odd fluids (Fig. 4).

In 2D atmospheric flows and confined plasmas, for instance, Rossby waves (also called drift waves) are at the origin of the arrest of an inverse cascade (Fig. 4b), at a scale *k*_Rh_ known as the Rhines scale^13–19^. This leads to the appearance of a pattern with characteristic scale *k*_Rh_ accompanied by a one-dimensionalization of the flow (Extended Data Fig. 2), eventually leading to mean flows known as zonal flows. Other waves, such as gravity waves in stratified flows, can play a similar part^44,45^. In contrast with the case of odd waves in 3D, there is no arrested direct cascade in these (quasi-)2D systems. In space plasma such as the solar corona (Fig. 4c), the existence of a ‘helicity barrier’ leading to the arrest of cascades has been proposed and traced to the change of nature of inviscid invariants. This mechanism is enabled by the existence of additional degrees of freedom in magnetohydrodynamics (MHD) compared with standard hydrodynamics. In the case of odd turbulence, the only inviscid invariants are energy and helicity (Methods), exactly as in standard turbulence. Beyond fluids, a weak turbulence theory for odd waves could also be applied, for instance, to optical or elastic turbulence^26–28^. In this case, arbitrary dispersion relations could be designed using metamaterials^9,43^, for example, by using a combination of so-called odd and even elastic moduli, which replace viscosities in elastodynamics^9^.

## Scale selection by mass cascades

Cascade-induced patterns can also occur in systems in which it is mass rather than energy that cascades (Fig. 4d). Mass cascades can, for instance, take place in the pulverization of objects into debris or the coalescence and breakup of droplets^23,25^. In this context, a cascade-induced scale selection would manifest in the selection of objects with a preferred scale that is neither the largest nor the smallest possible size. The existence of a steady state with such a characteristic scale can be observed in situations ranging from rain formation^24^ and smoke aerosols^25^ to active mixtures^46–51^. In the Methods, we present a minimal model of scale selection in the steady state of a mass cascade, in the spirit of shell models of turbulence^52^. The key idea is that large droplets (or clusters) tend to break up, whereas small ones tend to coalesce, similar to vortices in odd fluids: the rate of aggregation ${w}_{k}^{+}$ increases with *k* (Fig. 4d, red curve), whereas the rate of fragmentation ${w}_{k}^{-}$ decreases (blue curve). This can be captured within a population balance model that we analyse in the Methods using numerical simulations and analytical solutions. As shown in Extended Data Fig. 3, a preferential scale, that is neither the largest nor the smallest droplet size, emerges from the balance between these two physical processes, which play a similar part as the homochiral and heterochiral channels in odd fluid turbulence. This kind of scale selection can also occur in closed systems in which mass is neither injected nor removed (that is, with no net flux), such as in the arrested or interrupted coarsening of mixtures^46–51^.

## Conclusion

We have developed a theory of turbulent cascades modified by odd waves that captures how nonlinear scale selection emerges because of the arrest of the 3D direct and inverse cascades. Our work highlights the impact of waves in the fluid on eddy turbulence. Beyond fluid turbulence, similar mechanisms of scale selection may occur in domains ranging from wave turbulence in parity-violating optical media or solids with odd elasticity to mass cascades as well as cascades that occur in the time domain^53,54^.

## Methods

### Direct numerical simulations of the Navier–Stokes equation with odd viscosity

Direct numerical simulations of the Navier–Stokes equation with odd viscosity (equation (1)) are performed in a cubic box of size *L* = 2π with periodic boundary conditions (such that the smallest wavenumber is 2π/*L* = 1). Our results can be reproduced with any Navier–Stokes solver by including a modified Coriolis term modulated by *k*^2^ (or, equivalently, by a vector Laplacian for real-space-based methods) to account for odd viscosity. We use a pseudo-spectral method with Adams–Bashforth time-stepping and a 2/3-dealiasing rule^58^. Both normal and odd viscosities are integrated exactly using integrating factors. The forcing **f**(*t*, **k**) acts on a band of wavenumbers *k* ∈ [*k*_in_, *k*_in_ + 1] with random phases that are delta-correlated in space and time, ensuring a constant average energy injection rate *ϵ* = ⟨**u** ⋅ **f**⟩. It has a zero mean component ⟨**f**(*t*, **k**)⟩ = **0** and covariance $\langle {\bf{f}}(t,{\bf{k}})\cdot {\bf{f}}({t}^{{\prime} },{{\bf{k}}}^{{\prime} })\rangle ={\epsilon }\delta (t-{t}^{{\prime} })\delta ({\bf{k}}-{{\bf{k}}}^{{\prime} })$. The time-step is chosen to resolve the fastest odd wave with frequency ${\tau }_{{\rm{odd}},\max }^{-1}={\nu }_{{\rm{odd}}}{k}_{\max }^{2}$, where ${k}_{\max }$ is the highest resolved wavenumber in the domain. We find that stable integration requires a time-step $\Delta t\lesssim 0.1{\tau }_{{\rm{odd}},\max }$. A complete overview of the input parameters for the simulations in this work is provided in the Supplementary Information. Approximately 3 million CPU hours were required to perform the simulations underlying this work.

### Effect of odd waves on the nonlinear energy transfer

In this section, we describe how the waves induced by odd viscosity (odd waves) affect the nonlinear energy transfer. Our analysis closely follows that of rotating turbulence^2,4,36,59^.

#### Nonlinear energy transfer

Fourier-transforming the Navier–Stokes equation, multiplying with v^*^(*t*, **k**) (where the asterisk denotes complex conjugation), and adding the complex conjugate, we find the energy balance equation^2,4^

6 $${\partial }_{t}E=-2\nu {k}^{2}E-T+F$$

where *ν* = *η*/*ρ* is the kinematic viscosity, and in which

7 $$T({\bf{k}},t)={\rm{I}}{\rm{m}}\sum _{{\bf{k}}+{\bf{p}}+{\bf{q}}={\bf{0}}}{v}_{i}^{\ast }({\bf{k}},t){P}_{ij}({\bf{k}}){q}_{\ell }{v}_{\ell }^{\ast }({\bf{p}},t){v}_{j}^{\ast }({\bf{q}},t).$$

This term describes the nonlinear energy transfer between scales, whereas *F* = v^*^ ⋅ **f** corresponds to energy injection by the forcing term **f**. The term −2*νk*^2^*E* represents standard viscous dissipation. In equation (7), the sum runs on momenta **p** and **q** such that **k** + **p + q = 0**, and *P*_*ij*_(**k**) = *δ*_*ij*_ − *k*_*i*_*k*_*j*_/*k*^2^ is the projector on incompressible flows.

At first glance, equation (6) is left unchanged by odd viscosity, because of its non-dissipative nature. However, odd viscosity has indirect effects on the energy transfer (in the same way as the non-dissipative Coriolis force has an indirect effect on the energy transfer in rotating turbulence).

#### Odd waves

To see that, we first consider the linear and inviscid limit of the Navier–Stokes equation (1) (so we set *ν* = 0 and (**u** ⋅ **∇**)**u** = 0). As detailed in the section ‘Linear stability of the fluid and odd waves’ of the Supplementary Information (in which we consider a more general odd viscosity tensor), this equation has wave solutions of the form

8 $${\bf{v}}(t,{\bf{x}})={{\bf{h}}}^{\pm }({\bf{k}})\,{{\rm{e}}}^{{\rm{i}}{\omega }_{\pm }({\bf{k}})t+{\rm{i}}{\bf{k}}\cdot {\bf{x}}}+\,{\rm{c.c.}}$$

in which **h**^±^(**k**) = **e**(**k**) × (**k**/*k*) ± i**e**(**k**) with ${\bf{e}}({\bf{k}})={\widehat{{\bf{e}}}}_{z}\times {\bf{k}}/\parallel {\widehat{{\bf{e}}}}_{z}\times {\bf{k}}\parallel$ (ref. ^60^) with frequency

9 $${\omega }_{\pm }({\bf{k}})=\pm {\nu }_{{\rm{odd}}}{k}_{z}| k| .$$

Taking into account normal viscosity leads to an additional exponential decay of the waves with the rate −*νk*^2^ (Supplementary Information). In particular, we note that the linearized Navier–Stokes equation does not exhibit any linear instability. By construction, **k** ⋅ **h**^±^(**k**) = 0, so these modes represent incompressible flows. Furthermore, ${({{\bf{h}}}^{+}({\bf{k}}))}^{* }\cdot {{\bf{h}}}^{-}({\bf{k}})=0$ and ${({{\bf{h}}}^{\pm }({\bf{k}}))}^{* }\cdot {{\bf{h}}}^{\pm }({\bf{k}})=2$. Hence, odd waves provide an orthonormal basis for incompressible flows. As **k** × **h**^±^ = −*k***h**^±^, the basis functions have a well-defined helicity ∓1.

#### Decomposition of the energy transfer on odd waves

Expanding the velocity field as a superposition of helical waves

10 $${\bf{v}}(t,{\bf{x}})=\sum _{{\bf{k}}}\sum _{s=\pm }{v}_{s}(t,{\bf{k}}){{\bf{h}}}^{s}({\bf{k}}){{\rm{e}}}^{{\rm{i}}\omega t+{\rm{i}}{\bf{k}}\cdot {\bf{x}}}$$

in which ${v}_{s}^{* }(t,{\bf{k}})={v}_{s}(t,-{\bf{k}})$ to ensure the reality of **v**(*t*, **x**), the Navier–Stokes equation becomes

11 $${\partial }_{t}{v}_{{s}_{k}}=\sum _{\begin{array}{c}{\bf{k}}+{\bf{p}}+{\bf{q}}={\bf{0}}\\ {s}_{p},{s}_{q}=\pm \end{array}}{C}_{k| p,q}{{\rm{e}}}^{{\rm{i}}[\omega ({\bf{k}})+\omega ({\bf{p}})+\omega ({\bf{q}})]t}{v}_{{s}_{p}}^{* }{v}_{{s}_{q}}^{* }-\nu {k}^{2}{v}_{{s}_{k}}+{f}_{{s}_{k}}$$

in which we have used the short ${v}_{{s}_{k}}$ for ${v}_{{s}_{k}}(t,{\bf{k}})$, the term ${f}_{{s}_{k}}({\bf{k}})$ corresponds to the forcing term, and

12 $${C}_{k| p,q}=-\frac{1}{4}({s}_{p}p-{s}_{q}q){[({{\bf{h}}}^{{s}_{p}}({\bf{p}})\times {{\bf{h}}}^{{s}_{q}}({\bf{q}}))\cdot {{\bf{h}}}^{{s}_{k}}({\bf{k}})]}^{* }$$

satisfy *C*_*k*∣*p*,*q*_ = *C*_*k*∣*q*,*p*_.

#### Helicity and energy conservation of inviscid invariants

In terms of the components *v*_±_(**k**), energy and helicity, respectively, read^4,60^

13 $$E=\sum _{{\bf{k}}}(| {v}_{+}({\bf{k}}){| }^{2}+| {v}_{-}({\bf{k}}){| }^{2})$$

14 $$H=\sum _{{\bf{k}}}k(| {v}_{+}({\bf{k}}){| }^{2}-| {v}_{-}({\bf{k}}){| }^{2}).$$

A direct calculation shows that^36,60^

15 $${C}_{k| p,q}+{C}_{p| q,k}+{C}_{q| k,p}=0$$

and

16 $${s}_{k}k{C}_{k| p,q}+{s}_{p}p{C}_{p| q,k}+{s}_{q}q{C}_{q| k,p}=0$$

from which we deduce that energy and helicity are conserved when normal viscosity and the forcing can be neglected (*ν* = 0 and **f** = **0**), even if odd viscosities are present. In particular,

17 $${\partial }_{t}E(k)={v}_{{s}_{k}}^{* }{\partial }_{t}{v}_{{s}_{k}}+\,{\rm{c.c.}}$$

so using equation (11), we find (when *ν* = 0 and **f** = **0**)

18 $${\partial }_{t}E(k)=\sum _{\begin{array}{c}{\bf{k}}+{\bf{p}}+{\bf{q}}={\bf{0}}\\ {s}_{p},{s}_{q}=\pm \end{array}}{C}_{k| p,q}{{\rm{e}}}^{{\rm{i}}[\omega ({\bf{k}})+\omega ({\bf{p}})+\omega ({\bf{q}})]t}{v}_{{s}_{k}}^{* }{v}_{{s}_{p}}^{* }{v}_{{s}_{q}}^{* }+\,{\rm{c.c.}}\,$$

This equation shows that the nonlinear energy transfer *T*(**k**, *t*) in equation (7) is suppressed when averaged over long times compared to *ω*(**k**) + *ω*(**p**) + *ω*(**q**), unless this quantity vanishes exactly, as is the case for 2D modes (Fig. 2h, blue line, corresponding to modes with *k*_*z*_ = 0).

#### Resonant manifold

The 2D modes with *k*_*z*_ = 0 form a so-called slow manifold, or resonant manifold, that contributes to most of the nonlinear energy transfer. Furthermore, isolated triads with *k*_*z*_ ≠ 0 can also satisfy the resonance condition *ω*(**p**) + *ω*(**q**) + *ω*(**k**) = 0. In the case of rotating turbulence, resonant triads primarily transfer energy from the 3D modes to the quasi-2D slow manifold with *k*_*z*_ = 0, leading to an accumulation of energy in the slow manifold, enhancing the two-dimensionalization of the flow^4,33–36^. We expect a similar phenomenon to occur in the case of fluids with odd viscosity owing to its similarity to rotating fluids, as is also suggested by the two-dimensionalization observed in our numerical simulations. As a consequence, the effective spatial dimension of the system depends on the scale at which it is observed (such as in rotating turbulence or thick layers^4,35,61^). More insights may be obtained by developing a weak turbulence theory for odd waves, in the same spirit as for rotating flows (we refer to refs. ^26–28^ for more details on wave turbulence).

### Scaling relations and wavelength selection

#### Scaling relation for the energy spectrum

We first analyse the power spectrum, building on the phenomenological theory of ref. ^39^ (see ref. ^42^ for a review). This theory relies on the following hypotheses: (1) energy is conserved away from injection and dissipative scales; (2) the cascade is local, which means that different length scales are coupled only locally (for example, very large scales are not directly coupled to very small scales); and (3) the rate of energy transfer *ε*(*k*) from scales higher than *k* to scales smaller than *k* is directly proportional to the triad correlation time *τ*_3_. Because of hypotheses 1 and 2, the rate of energy transfer *ε*(*k*) is constant across the scales (that is, does not depend on *k*) and can be identified with the energy dissipation rate *ϵ*. Moreover, because of hypothesis 2, *ε*(*k*) should depend only on local quantities *k* and *E*(*k*), in addition to *τ*_3_(*k*). Therefore, using hypothesis 3, we write

19 $${\epsilon }=\varepsilon (k)=A{\tau }_{3}(k){k}^{\alpha }{E}^{\beta }$$

where *A* is a constant. The exponents are found using dimensional analysis (with [*E*] = *L*^3^*T*^−2^, [*ϵ*] = *L*^2^*T*^−3^, [*k*] = *L*^−1^, [*τ*_3_] = *T*), which yields *α* = 4 and *β* = 2.

In Fig. 2, we argue that the eddy turnover time *τ*_E_ is the relevant timescale when *k* ≪ *k*_odd_ (so we set *τ*_3_ = *τ*_E_ in the expression above), whereas the frequency of odd waves *ω* is the relevant timescale when *k* ≫ *k*_odd_ (so we set *τ*_3_ = *ω*^−1^). The dispersion relation of odd waves is computed in the Supplementary Information and given in equation (9). The eddy turnover time is *τ*_E_(*k*) = [1/*k*]/*v*_*k*_. As *E*(*k*) is the shell-average of *v*_*k*_, we have dimensionally $E(k)\propto {v}_{k}^{2}/k$, so *v*_*k*_ = [*kE*(*k*)]^1/2^. Putting everything together, we end up with equations (3) and (4) of the main text.

#### Scaling relation for *k*_c_

For the condensation of the forward energy flux, the collapse of numerical results indicates that it can be described by a master scaling law

20 $$\frac{E(k)}{{E}_{0}(k)}\propto \left\{\begin{array}{ll}1 & \,\mathrm{for}\,\,k\ll {k}_{{\rm{odd}}},\\ {\left(k/{k}_{{\rm{odd}}}\right)}^{s} & \,\mathrm{for}\,\,k\gg {k}_{{\rm{odd}}},\end{array}\right.$$

Using the Kolmogorov spectrum for the case without odd viscosity, *E*_0_(*k*) ∝ *ϵ*^2/3^*k*^−5/3^, we find for the energy spectrum

21 $$E(k)\propto \left\{\begin{array}{ll}{{\epsilon }}^{2/3}{k}^{-5/3} & \,\mathrm{for}\,\,k\ll {k}_{{\rm{odd}}},\\ {{\epsilon }}^{2/3}{k}^{-5/3}{\left(k/{k}_{{\rm{odd}}}\right)}^{s} & \,\mathrm{for}\,\,k\gg {k}_{{\rm{odd}}}.\end{array}\right.$$

Using the scaling argument of the previous section (see equations (3) and (4)), we find *s* = 2/3, which is compatible with the numerical results. This scaling continues until dissipation saturates the condensation. We can thus estimate the location of the condensation peak *k*_c_ from the balance between injection and dissipation. Neglecting contributions to the dissipation from wavenumbers *k* < *k*_odd_ (where there is no meaningful change from Kolmogorov scaling), we obtain

22 $${\epsilon }\propto {\int }_{{k}_{{\rm{o}}{\rm{d}}{\rm{d}}}}^{{k}_{{\rm{c}}}}\nu {k}^{2}E(k){\rm{d}}k.$$

Assuming *k*_odd_ ≪ *k*_c_, this yields

23 $${\epsilon }\propto \nu \,{{\epsilon }}^{2/3}\,{k}_{{\rm{c}}}^{4/3}{({k}_{{\rm{c}}}/{k}_{{\rm{o}}{\rm{d}}{\rm{d}}})}^{s},$$

resulting in the scaling relation for the peak condensation

24 $${k}_{{\rm{c}}}\propto {\left({{\epsilon }}^{1/3}{\nu }^{-1}{k}_{{\rm{odd}}}^{s}\right)}^{\frac{1}{4/3+s}}\propto {\left({k}_{\nu }^{4/3}{k}_{{\rm{odd}}}^{s}\right)}^{\frac{1}{4/3+s}},$$

where in the last relation, we substituted the normal Kolmogorov wavenumber *k*_*ν*_ ∝ *ϵ*^1/4^*ν*^−3/4^.

For *s* = 2/3, we find

25 $${k}_{{\rm{c}}}\propto {\left({k}_{\nu }^{4/3}{k}_{{\rm{odd}}}^{2/3}\right)}^{1/2}\propto {{\epsilon }}^{1/4}{\nu }^{-1/2}{\nu }_{{\rm{odd}}}^{-1/4}$$

as quoted in the main text.

#### Estimation of the height for the peak

The mechanism of non-dissipative arrest analysed in this work is reminiscent of but distinct from the bottleneck effect^62–67^ generated by the usual viscosity.

**Extended Data Fig. 1:**
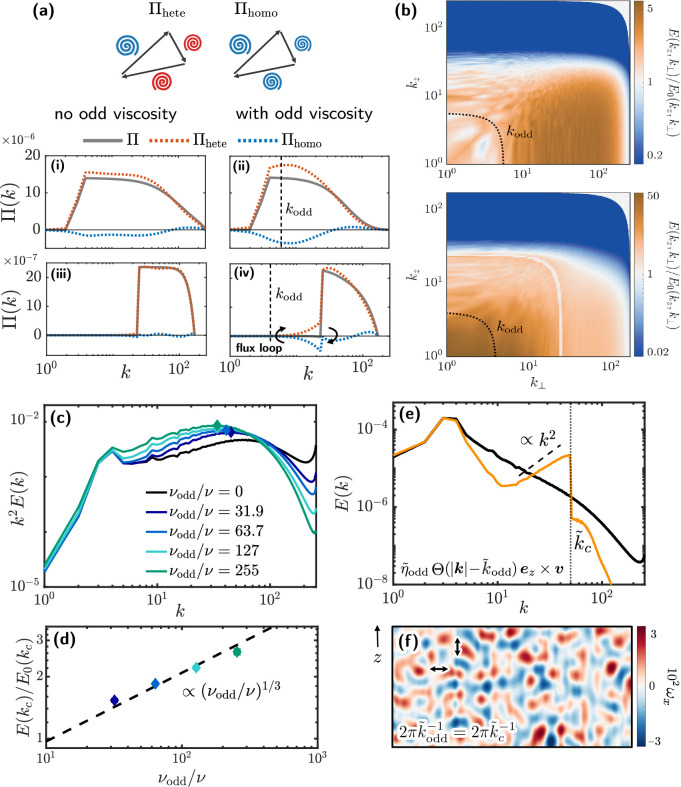
Arrest of turbulent cascades in numerical simulations. (a) The total energy flux Π(*k*) is decomposed into heterochiral Π_hete_(*k*) and homochiral Π_homo_(*k*) components for direct cascading cases (i,ii) and inverse cascading cases (iii,iv). The cases without odd viscosity (i,iii) are compared to the cases with odd viscosity *ν*_odd_/*ν* = 255 (ii) and *ν*_odd_/*ν* = 212 (iv). Odd viscosity enhances the homochiral manifold that predominantly cascades inversely, which is in turn balanced by an increased heterochiral flux. For the inverse cascading cases this leads to a flux loop condensate state with vanishing net flux. In (iii, iv) hyperdissipation is used to mimic increased scale separation. See also the section *Helical decomposition* in the SI. (b) The anisotropic kinetic energy spectrum *E*(*k*_*z*_, *k*_⊥_) with odd viscosity normalized by the case without odd viscosity *E*_0_(*k*_*z*_, *k*_⊥_) for the forward cascading case with *ν*_odd_/*ν* = 255 (top panel) and the inverse cascading case with *ν*_odd_/*ν* = 212 (bottom panel). Both panels indicate the regions in *k*-space where the energy condensation due to odd viscosity occurs. (c) In order to determine the characteristic wavelengths in the vorticity field for the direct cascading case, we compute the vorticity spectrum ∥***ω***(***k***)∥^2^ as *k*^2^*E*(*k*). Without odd viscosity, the maximum of the spectrum is close to the dissipative scale. When odd viscosity is present, a stronger peak emerges in the spectrum as a consequence of the spectral condensation at intermediate scales, evidencing the wavelength selection. (d) The lozenges give the value of *h* ≡ *E*(*k*_c_)/*E*_0_(*k*_c_) obtained from the numerical simulations, and are compared with the predicted scaling $h\propto {({\nu }_{{\rm{odd}}}/\nu )}^{1/3}$ (black dashed line). (e) We demonstrate the kinetic energy spectrum *E*(*k*) for the case of a sharp transition from a direct cascade at small *k* to an inverse cascade at large *k*, modeled as a step function of odd viscosity, stepping at ${\mathop{k}\limits^{ \sim }}_{{\rm{o}}{\rm{d}}{\rm{d}}}$ (orange), compared to the case without odd viscosity (black). Here, Θ is the Heaviside step function. The sharp transition leads to a sharp condensation at ${\mathop{k}\limits^{ \sim }}_{c}\equiv {\mathop{k}\limits^{ \sim }}_{{\rm{o}}{\rm{d}}{\rm{d}}}$ and a diffusive equipartitioned scaling ∝*k*^2^ to the left of it. The resulting pattern in *ω*_*x*_ is shown in (f), with typical wavelength ${\widetilde{k}}_{c}^{-1}={\widetilde{k}}_{{\rm{odd}}}^{-1}$ in both the horizontal and vertical directions. See also the section *Odd hyperviscosity* in the SI.

A coarse estimate of the height of the peak in *E*(*k*)/*E*_0_(*k*) can be obtained by evaluating equation (4) (to get *E*(*k*)) and equation (3) (to get *E*_0_(*k*)) at *k* = *k*_c_ given by equation (5), yielding $$h\equiv E({k}_{{\rm{c}}})/{E}_{0}({k}_{{\rm{c}}})\,\propto $$$${({\nu }_{{\rm{odd}}}/\nu )}^{1/3}$$ (see Extended Data Fig. 1d for a comparison with numerical data). Notably, this suggests that *h* depends on only the ratio of odd to normal viscosity. We also note that *h* increases as normal viscosity *ν* decreases (that is, when the Reynolds number increases), in contrast with the bottleneck effect due to dissipative viscosity^62–67^ in which the magnitude of the effect decreases as viscosity decreases.

#### Wavelength selection

In Extended Data Fig. 1c, we plot an estimate of the power spectrum of the vorticity, evidencing wavelength selection in the vorticity. This suggests that the characteristic wavelength 2π/*k*_c_ should be directly visible in snapshots of the vorticity field. This can be seen in Fig. 3b. The width of the peak leads to a wide distribution of structure sizes in the image.

We expect the wavelength selection mechanism due to the arrested cascade to persist at arbitrarily long times and to resist small perturbations, in contrast with metastable patterns arising from kinetic effects^68^ in which the system resides in metastable states for long but finite periods (see Supplementary Information for convergence plots).

The wavelength selection mechanism we have described can be compared with that in active turbulence, for instance in bacterial suspensions and self-propelled colloids^69–74^. In active turbulence, however, it has been reported that there is no energy transfer across scales (and hence no cascade): energy is typically dissipated at the same scale as it is injected, and it is believed that the wavelength selection is the result of a scale-by-scale balance (see, for instance, Figs. 3d and 4g and sections 3.2.2 and 4.2.3 in ref. ^71^ and references therein). We note, however, that finite energy fluxes have been reported in certain cases^56,75–80^.

In these systems, wavelength selection has been described as the result of a Swift–Hohenberg-type term included in the stress tensor (leading to a finite-wavelength linear instability), to which noise is added^71^. By contrast, cascade-induced pattern formation cannot be directly traced to a linear instability of Navier–Stokes equation (1) (see section ‘Effect of odd waves on the nonlinear energy transfer’ as well as Supplementary Information section ‘Linear stability of the fluid and odd waves’ for a linear stability analysis). The linear stability analysis does not predict any instability, neither to a stable branch with a particular wavelength nor to an unstable branch that could itself bifurcate to the state of interest as part of a subcritical bifurcation.

An analogy with similar situations such as Rossby and drift wave turbulence^81–86^ and laminar and turbulent patterns in wall-bounded shear flows^87–90^ suggests that the wavelength selection may be described by considering the linear stability of the statistically averaged Navier–Stokes equation, for instance, using an appropriate turbulence closure model.

#### Anisotropic energy spectra

In line with the inherent symmetry of the system, we now consider cylindrically averaged energy spectra *E*(*k*_⊥_, *k*_*z*_), which distinguish the horizontal (perpendicular) directions from the vertical direction^91–94^. To reveal in which part of the *k*-space the energetic condensation occurs, we compute the cylindrically averaged spectrum of the cases with odd viscosity normalized by the spectrum of the reference case without odd viscosity (Extended Data Fig. 1b). Starting with the direct cascading case in Extended Data Fig. 1b (top panel), we see that indeed the flow remains mostly 3D isotropic for *k* < *k*_odd_ and then proceeds to condensate anisotropically into the low-*k*_*z*_ manifold because of the quasi-2-dimensionalization effect of the odd viscosity. As detailed in the main text, the condensation is saturated by dissipation, leading to a peak condensation wavelength *k*_*c*_, which is thus primarily visible in the perpendicular directions because of the anisotropic condensation. The dominant vertical scale hence remains closer to *k*_odd_. This leads to a crude estimate for the aspect ratio *γ* of the features in the pattern produced by the odd viscosity as

26 $$\gamma =\frac{{k}_{c}}{{k}_{{\rm{odd}}}}\propto {\nu }^{-1/2}{\nu }_{{\rm{odd}}}^{1/2}.$$

For the case presented in Fig. 3b, this leads to an aspect ratio *γ* ≃ 3.

For the inverse cascading case (Extended Data Fig. 1b, bottom panel), we again observe anisotropic condensation in the region *k* > *k*_odd_. In the region *k* < *k*_odd_, however, the kinetic energy for the case with odd viscosity is larger than the case without odd viscosity, as indicated in dark orange. This is because in this range, we expect the same diffusive equipartitioned scaling *E*(*k*) ∝ *k*^2^ for both cases with and without odd viscosity, and there is no active dissipative mechanism to deplete the excess energy that has accumulated at higher wavenumbers in the case with odd viscosity.

### Experimental considerations

In this section, we discuss the conditions required to observe the wavelength selection described in the main text in a fluid with odd viscosity. In short, we expect this effect to occur, for instance, in a fluid of self-spinning particles large enough to be inertial (not overdamped).

First, the Reynolds number Re = *UL*/*ν* has to be large enough. This puts constraints on the viscosity *ν* of the fluid, the details of which depend on the experimental setup considered. The current experimental systems we are aware of in which explicit measurements of odd viscosities were reported (active spinning colloids^32^, magnetized graphene^10^ and magnetized polyatomic gases^31,95^) are all in a regime in which the nonlinear advective term in the Navier–Stokes equation can be neglected, either because *ν* is large enough or for geometric reasons; effectively, Re ≪ 1. Note also that experimental instances of (especially 2D) odd fluids may include a substrate, on top of which the active particles move. This can lead to the addition of an effective drag force −*γ***v** in the Navier–Stokes equation describing the odd fluid made of these particles. If such a term is large, it would prevent the existence of an inertial regime, and probably spoil the phenomenology discussed here.

Second, the ratio *ν*^odd^/*ν* has to be large enough for the effect to be visible. When *ν*^odd^ ≲ *ν*, energy is dissipated as soon as, or before any effect of odd waves can arise. Henceforth, observing the effects of odd waves on turbulence would require *ν*^odd^ > *ν*. Odd viscosities (*ν*^odd^ ≠ 0) typically arise in systems breaking time-reversal and inversion symmetry at the microscopic scale^9,96,97^. They have been experimentally measured in polyatomic gases under magnetic fields^31,95^, spinning colloids^32^ and magnetized electron fluids^10^. They have also been predicted in systems, including fluids under rotation^98^, magnetized plasma^11,99,100^, quantum fluids^96,101–103^, vortex matter^104^, sheared granular gases^105^, assemblies of spinning objects^69,106–117^ and circle swimming bacteria^118,119^. In the systems mentioned above, in which experimental measurements of odd viscosity have been reported, *ν*_odd_/*ν* reaches at most 1/3 (in active spinning colloids^32^ and magnetized graphene)^10^. From a theoretical point of view, the ratio *ν*_odd_/*ν* is expected to increase linearly with the time-reversal breaking field. For instance, ideal vortex fluids are predicted to have a finite *ν*_odd_ but a vanishing *ν* (ref. ^104^), leading to an infinite value of *ν*_odd_/*ν*. Kinetic theory calculations for magnetized plasma (ref. ^99^, section 19.44) predict *ν* = *ν*_0_/[1 + *x*^2^] and *ν*_odd_ = *ν*_0_*x*/[1 + *x*^2^] in which *x* = 2*ωτ* with *τ* is a collision time and *ω* ∝ *B* is a frequency proportional to the magnetic field *B*, whereas *ν*_0_ is the value of normal shear viscosity when *B* = 0. Similarly, kinetic theory in rotating gases leads to an identical result in which *x* ∝ *Ω* is proportional to the rotation speed^98^. In electron gases in graphene, experiments have been performed that validate these theoretical calculations^10^ (with *x* = *B*/*B*_0_, where *B*_0_ is a reference magnetic field). This results in a ratio *ν*_odd_/*ν* = *x* ∝ *B*. Likewise, in active fluids, theoretical works suggest that *ν*_odd_ is proportional to the rotation speed of the spinning particles^106^.

### Rossby and drift wave turbulence

**Extended Data Fig. 2:**
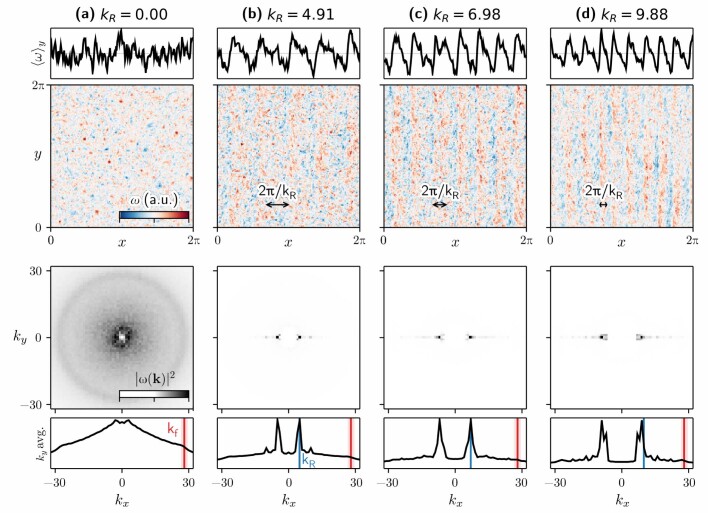
Rossby/drift wave turbulence. Simulations of Eq. (27) describing Rossby/drift wave turbulence demonstrates one-dimensionalization of the 2D flow and the appearance of a pattern with characteristic scale given by the Rhines scale 1/*k*_R_^13–19,127^. Each column shows, from top to bottom: (i) the vorticity averaged along the y direction at final time, (ii) the vorticity field at final time, (iii) the power spectrum of the vorticity averaged over the last 1/6 of the simulation and (iv) the *k*_*y*_ average of this quantity. The equation is integrated using the pseudospectral solver Dedalus^128^ on a *L* × *L* square domain with size *L* = 2π discretized with *N* = 256 Fourier harmonics per dimension using a 3rd-order 4-stage Diagonally Implicit/Explicit Runge-Kutta scheme (RK433 in Dedalus)^166^ with an adaptive timestep for 1500 simulation time units. The forcing is taken to be a Gaussian random field concentrated on a ring of radius *k*_f_ = 28 (red line) and bandwidth *k*_fw_ = 1.5 (light red rectangle) in Fourier space, scaled by the forcing rate *ϵ* = 0.001. We take a linear drag *α* = 0.01, a viscosity *ν* = 0.00001. The *β* parameter is (a) *β* = 0, (b) *β* = 20, (c) *β* = 40, (d) *β* = 80, leading to the measured values of the Rhines wavenumber ${k}_{{\rm{R}}}=1/2\sqrt{\beta /{U}_{{\rm{rms}}}}$ given in the figure (blue line in the bottom plots) in which *U*_rms_ is obtained from the measured energy spectrum.

Extended Data Fig. 2 shows examples of simulations of the Rossby and drift wave turbulence mentioned in Fig. 4b. A brief review is contained in the Supplementary Information, and we refer the reader to refs. ^13–19,93,120–127^ for more details. In the figure, we simulate the Charney–Hasegawa–Mima (CHM) equation^93,120–126^

27 $${\partial }_{t}\omega +J(\psi ,\omega )+\beta {\partial }_{x}\psi =-\alpha \omega +\nu \Delta \omega +{f}_{\omega }$$

in which *J*(*a*, *b*) = (∂_*x*_*a*)(∂_*y*_*b*) − (∂_*y*_*a*)(∂_*x*_*b*), *ω* = Δ*ψ* and *ψ* is the stream function, defined such that the velocity field is **v** = −*ϵ* ⋅ ∇*ψ* (*ϵ* is the 2D Levi-Civita symbol). The parameter *β* represents the gradient of the Coriolis force in a *β*-plane approximation; *α* represents large-scale friction and *ν* is viscosity, whereas *f*_*ω*_ is a vorticity forcing. Simulations are performed using the open-source pseudo-spectral solver Dedalus^128^.

Note that in Rossby wave turbulence, the only exact inviscid invariants are energy and helicity. However, it has been established that a quantity dubbed zonostrophy evolves slowly enough to be considered as an invariant for practical purposes^13,93,129–131^. This raises the question of whether such an adiabatic invariant may exist for odd turbulence, and whether it can predict the direction of the cascades (see refs. ^4,132^ for discussions of the relation between inviscid invariants and the direction of turbulent cascades).

### Minimal model of mass cascade with scale selection

In this section, we consider a simple model of the mass cascade that exhibits wavelength selection.

Mass cascades can, for instance, occur in the pulverization of objects into debris or in the coalescence and breakup of droplets^23,133–138^. These processes can be modelled by the aggregation and fragmentation of clusters composed of monomers linked together: two clusters that collide may merge into a larger cluster; and a given cluster may split into smaller ones, spontaneously or on collision. The mean-field kinetics of these processes is described by a population balance equation generalizing the so-called Smoluchowski equation^23,139–141^ that can exhibit scale-invariant cascades, similar to that present in the Navier–Stokes equation^142–144^. This kinetic equation may describe two classes of situations: (1) closed systems in which mass is conserved and (2) open systems in which particles are injected and removed from the system. Case 1 may somehow be compared with freely decaying turbulence, whereas case 2 may be compared with driven turbulence in which energy is injected and dissipated.

We expect that the balance between aggregation and fragmentation will lead to a preferred size if large clusters tend to break up, whereas small clusters tend to coalesce. Such a preferred size should manifest as a peak in the distribution of aggregate sizes. Such a peak has been reported, for instance, in the case of raindrop sizes^24,145,146^, in which the distribution originates from complex mechanisms, including air turbulence and fluid fragmentation^147–152^.

In our toy model, we consider clusters *M*_*n*_ composed of 2^*n*−1^ monomers *M*_1_, with *n* = 1, …, *N*. This is reminiscent of what is done in shell models of turbulence^52^, in which the wavenumbers are chosen in geometric progression. We assume that (1) there are interactions only between clusters of the same size and (2) there is a maximum cluster size *N*. The first assumption ensures that the mass fluxes are local, and the second enables us to consider a finite number of equations. We include a constant source of monomers, and a sink that removes the largest clusters *M*_*N*_. In the case of raindrops in a cloud, for instance, the source may describe the condensation of droplets from vapour, and the sink may describe the precipitation of large droplets out of the cloud. The model is summarized by the set of reactions

28a $$\varnothing \,\mathop{\longrightarrow }\limits^{{J}_{{\rm{i}}{\rm{n}}}}\,{M}_{1}$$

28b $$2\,{M}_{n}\mathop{\mathop{\rightleftharpoons }\limits^{{k}_{n}^{+}}}\limits_{{k}_{n+1}^{-}}{M}_{n+1}$$

28c $${M}_{N}\,\mathop{\longrightarrow }\limits^{{J}_{{\rm{o}}{\rm{u}}{\rm{t}}}}\,\varnothing $$

in which *M*_*n*_ (*n* = 1, …, *N*) represents a cluster of size 2^*n*−1^ (*M*_1_ represents a monomer), and *J*_in_, *J*_out_ and ${k}_{n}^{\pm }$ are the rates of the corresponding reactions.

The number densities *c*_*n*_ of clusters then follow the dynamical equation

29 $$\frac{{\rm{d}}{c}_{n}}{{\rm{d}}t}=2{k}_{n+1}^{-}{c}_{n+1}-{k}_{n}^{-}{c}_{n}+\frac{1}{2}{k}_{n-1}^{+}{c}_{n-1}^{2}-{k}_{n}^{+}{c}_{n}^{2}+{J}_{{\rm{ext}}}$$

where

30 $${J}_{{\rm{ext}}}={\delta }_{n,1}{J}_{{\rm{in}}}-{\delta }_{n,N}{J}_{{\rm{out}}}{c}_{N}$$

in which it is implied that *c*_*n*_ ≡ 0 for *n* < 1 and *n* > *N*.

We can also consider the mass density *ρ*_*n*_ = 2^*n*−1^*m*_0_*c*_*n*_, in which *m*_0_ is the mass of a monomer. Multiplying equation (29) with 2^*n*−1^*m*_0_, we find that the terms with prefactors ${k}_{n}^{\pm }$ cancel as in a telescoping series. This manifests that equation (29) with *J*_in_ = *J*_out_ = 0 conserves mass. It is therefore convenient to introduce the fluxes

31 $${J}^{+}(n)=-{\int }_{1}^{n}\,d{n}^{{\prime} }\left[\frac{1}{2}{k}_{{n}^{{\prime} }-1}^{+}{c}_{{n}^{{\prime} }-1}^{2}-{k}_{{n}^{{\prime} }}^{+}{c}_{{n}^{{\prime} }}^{2}\right]$$

and

32 $${J}^{-}(n)=-{\int }_{1}^{n}\,d{n}^{{\prime} }[2{k}_{{n}^{{\prime} }+1}^{-}\,{c}_{{n}^{{\prime} }+1}-{k}_{{n}^{{\prime} }}^{-}\,{c}_{{n}^{{\prime} }}]$$

corresponding to the reactions with rates ${k}_{n}^{\pm }$, and such that

33 $$\frac{{\rm{d}}{c}_{n}}{{\rm{d}}t}=-{\partial }_{n}[\,{J}^{+}(n)+{J}^{-}(n)]+{\delta }_{n,1}{J}_{{\rm{in}}}-{\delta }_{n,N}{J}_{{\rm{out}}}{c}_{N}.$$

To induce wavelength selection, we choose particular forms for ${k}_{n}^{\pm }$. The basic idea is the forward flux ${k}_{n}^{+}$ should decrease with *n*, whereas the backward flux ${k}_{n}^{-}$ should increase with *n*. Experimentation suggests that various strictly increasing functions of (*N* − *n*)/(*N* − 1) and (*n* − 1)/(*N* − 1), respectively, lead to similar results. We choose

34 $${k}_{n}^{+}={\kappa }_{0}^{+}+{\kappa }_{1}^{+}\frac{N-n}{N-1}\,{\rm{and}}\,{k}_{n}^{-}={\kappa }_{0}^{-}+{\kappa }_{1}^{-}\frac{n-1}{N-1}.$$

**Extended Data Fig. 3:**
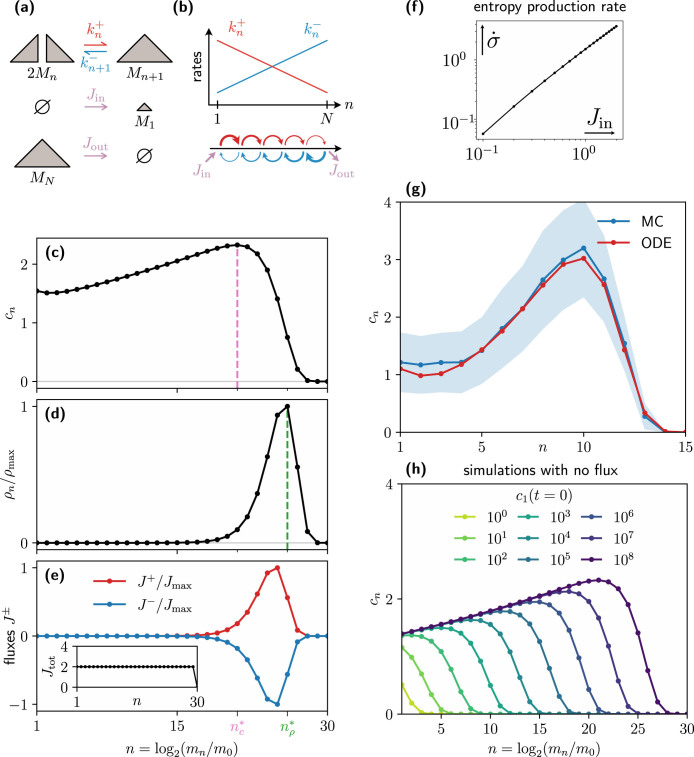
Minimal model of mass cascade with scale selection. (a) In the model, two clusters *M*_*n*_ of size 2^*n*−1^ can merge into a cluster *M*_*n*+1_ of size 2^*n*^. This aggregation process occurs with a rate ${k}_{n}^{+}$. Conversely, a cluster *M*_*n*+1_ can split into two clusters *M*_*n*_ (except for monomers *M*_1_). This fragmentation process occurs with a rate ${k}_{n+1}^{-}$. (b) The rates ${k}_{n}^{\pm }$ of aggregation/fragmentation depend on the size of the cluster, so that (i) large clusters are more likely to fragment that small ones (blue curve) and (ii) small clusters are more likely to aggregate than large ones (red curve). (c-e) Equations (29) are numerically solved starting from the initial condition *c*_*n*_ = 0 for all *n*. We have set ${\kappa }_{0}^{\pm }=2$ and ${\kappa }_{1}^{\pm }=1$, as well as *J*_in_ = *J*_out_ = 1. The number distribution *c*_*n*_ is plotted in panel c, while the mass distribution *ρ*_*n*_ = 2^*n*−1^*m*_0_*c*_*n*_ (normalized by its maximum value) is plotted in panel d. In panel e, we show the fluxes ${J}_{n}^{+}$ (red) and ${J}_{n}^{-}$ (blue) defined in Eqs. (31)–(32). The inset of panel e shows the total flux ${J}_{n}={J}_{n}^{+}+{J}_{n}^{-}$, which is constant away from the boundaries. (f) Entropy production in the mass cascade. We plot the rate of entropy production (computed within the mean-field model) as a function of the flux *J*_in_ through the system, in logarithmic scale. (As the distribution is stationary, there is a constant flux equal to the input flux). We have set $N=15,{\kappa }_{0}^{\pm }=0.1,{\kappa }_{1}^{\pm }=1,{J}_{{\rm{out}}}=1$ and *c*_*n*_(*t* = 0) = 0 for all *n*. (g) Comparison between mean-field and Monte-Carlo simulations. The red curve (labelled ODE) shows the mean-field solution of Eq. (29), while the blue curve (labelled MC) shows the solution of the kinetic Monte-Carlo simulations (average plus or minus half a standard deviation over 1000 samples). We have set $N=15,{\kappa }_{0}^{\pm }=0.1,{\kappa }_{1}^{\pm }=1,{J}_{{\rm{in}}}={J}_{{\rm{out}}}=1$ and *c*_*n*_(*t* = 0) = 0 for all *n*. (h) Equations (29) with no influx and outflux (*J*_in_ = *J*_out_ = 0) are numerically solved starting from the initial condition with only monomers *c*_*n*_(*t* = 0) = *c*_0_(0)*δ*_*n*,0_, with different values of the number of monomers *c*_0_(0). We observe that (i) a peak in the steady-state distribution only arises when the initial number of monomers *c*_0_(0) is large enough and (ii) the position of the peak moves as *c*_0_(0) increases. We have set ${\kappa }_{0}^{\pm }=2$ and ${\kappa }_{1}^{\pm }=1$.

Equation (29) is then solved starting from the initial condition *c*_*n*_ = 0 for all *n* using DifferentialEquations.jl (ref. ^153^) with a fourth-order A-stable stiffly stable Rosenbrock method (Rodas4P) until a steady state is reached. The resulting steady state is shown in Extended Data Fig. 3. In Extended Data Fig. 3c, we observe that the density *c*_*n*_ is peaked at an intermediate value ${n}_{c}^{* }$ (pink dashed line), which is neither the maximum cluster size *N*, nor the monomer size 1, demonstrating wavelength selection. Similarly, Extended Data Fig. 3d shows that the mass density *ρ*_*n*_ is peaked around a (different) scale ${n}_{\rho }^{* }$ (red dashed line). As we have considered a mean-field description that does not take space into account, there is no proper pattern-only wavelength selection.

We observe in Extended Data Fig. 3e that the flux *J*_tot_ ≡ *J*^+^ + *J*^−^ (black curve in inset) is constant and nonzero for 1 < *n* < *N*. In 1D, the existence of a steady state is equivalent to a constant flux. (Note that certain models of aggregation–fragmentation may exhibit oscillations, that is, limit cycles instead of fixed points^154,155^). The total flux can be decomposed into the forward flux *J*^+^ associated with reactions with rates ${k}_{n}^{+}$ and the backward flux *J*^−^ associated with reactions with rates ${k}_{n}^{-}$, respectively, defined in equations (31) and (32), and plotted in Extended Data Fig. 3e (red and blue curves, respectively).

In Extended Data Fig. 3h, we analyse the initial value problem obtained by setting *J*_in_ = *J*_out_ = 0 in equation (29). An exact solution of this model is given in the Supplementary Information. Wavelength selection may occur, although there is no net flux. This can be compared with the arrest of coarsening that can arise in mixtures and similar mass-conserving systems, even if the mass is not injected and removed from the system^46–51,156–158^. We also observe that wavelength selection occurs only when the total number of monomers is large enough, which is reminiscent of what happens in so-called beam self-cleaning in optics, in which light in an optical waveguide at sufficiently high power may undergo a nonlinear redistribution of the mode powers that favours the fundamental, similar to an inverse cascade^159^.

Equation (29) describes the mean-field dynamics of the reactions (28). To check whether the effect is still present beyond mean field, we solve the corresponding Doob–Gillespie kinetic Monte Carlo problem using the package Catalyst.jl (refs. ^153,160^). The result of the simulation is shown in Extended Data Fig. 3g, and compared with mean-field simulations, with excellent agreement.

Finally, we discuss the rate of entropy production in the system. To do so, it is convenient to introduce the rates ${k}^{+,n}={k}_{n}^{+}/2$ and ${k}^{-,n}={k}_{n+1}^{-}$ to match the notations used in the literature on chemical reaction networks^161–164^. We identify the forward and backward fluxes corresponding to the reaction with rates *k*^±,*n*^ as ${J}^{+,n}={k}^{+,n}{c}_{n}^{2}$ and *J*^−,*n*^ = *k*^−,*n*^*c*_*n*+1_. The rate of entropy production corresponding to the reaction is then ${\mathop{\sigma }\limits^{.}}_{n}=({J}^{+,n}-{J}^{-,n})\log ({J}^{+,n}/{J}^{-,n})$ (refs. ^161–164^). We can then evaluate this quantity and the total rate of entropy production $\mathop{\sigma }\limits^{.}={\sum }_{n}{\mathop{\sigma }\limits^{.}}_{n}$ from the steady-state distributions *c*_*n*_ obtained numerically (Extended Data Fig. 3f). The rate of entropy production vanishes when the system is isolated (*J*_in_ = *J*_out_ = 0), and increases as a function of the flux going through the system (which is equal to *J*_in_ as long as there is a stationary state).

## Online content

Any methods, additional references, Nature Portfolio reporting summaries, source data, extended data, supplementary information, acknowledgements, peer review information; details of author contributions and competing interests; and statements of data and code availability are available at 10.1038/s41586-024-07074-z.

### Supplementary information

Supplementary Information This file contains detailed derivations and explanations.

Supplementary Video 1 Comparison between odd turbulence and normal turbulence showing 3D scans of the snapshot from which the slices in Fig. 3a,b are extracted.

## Data Availability

The data generated during the course of this study is available on *Zenodo* at 10.5281/zenodo.10371195 (ref. ^165^).

## Code availability

The code used for processing the data, generating the figures and for the mass cascade and Rossby wave simulations as well as an executable for the DNS are available on *Zenodo* (10.5281/zenodo.10371195) under the 2-clause BSD licence (ref. ^165^).
