# Supplementary material for: Pattern formation by turbulent cascades
Source: Nature. 2024 Mar 20;627(8004):515–21. doi: 10.1038/s41586-024-07074-z (PMC10954557; doi:10.1038/s41586-024-07074-z)
Supplement: Supplementary file 1 — This file contains detailed derivations and explanations. [file 41586_2024_7074_MOESM1_ESM.pdf]

nature portfolio

https://doi.org/10.1038/s41586-024-07074-z

Supplementary information

Pattern formation by turbulent cascades

In the format provided by the authors and unedited

SI: Pattern formation by turbulent cascades

Xander M. de Wit,[1] Michel Fruchart,[2,3] Tali Khain,[3] Federico Toschi,[1,4] and Vincenzo Vitelli[3,5]

[1] *Department of Applied Physics and Science Education, Eindhoven University of Technology, 5600 MB Eindhoven, Netherlands*
[2] *Gulliver, ESPCI Paris, Université PSL, CNRS, 75005 Paris, France*
[3] *James Franck Institute, The University of Chicago, Chicago, IL 60637, USA*
[4] *CNR-IAC, I-00185 Rome, Italy*
[5] *Kadanoff Center for Theoretical Physics, The University of Chicago, Chicago, IL 60637, USA*

Navier-Stokes equation with odd viscosity

In this section, we describe the most general odd viscosity terms in a 3D incompressible fluid with cylindrical symmetry. In an incompressible fluid, any gradient term in the Navier-Stokes equation can be absorbed in the pressure, without modifying the flow. A generic cylindrically symmetric viscosity tensor can have eight independent non-dissipative (odd) viscosities [1]. Only two independent odd viscosity terms remain in the incompressible Navier-Stokes equation:

$$\rho_0 D_t \boldsymbol{v} = -\nabla P + \eta \Delta \boldsymbol{v} + \eta_1^{\mathrm{o}} \begin{bmatrix} (\partial_x^2 + \partial_y^2) v_y \\ -(\partial_x^2 + \partial_y^2) v_x \\ 0 \end{bmatrix} + \eta_2^{\mathrm{o}} \begin{bmatrix} -\partial_z^2 v_y - \partial_y \partial_z v_z \\ \partial_z^2 v_x + \partial_x \partial_z v_z \\ \partial_z (\partial_y v_x - \partial_x v_y) \end{bmatrix} \tag{S.1}$$

The forces due to the remaining odd viscosity coefficients can be expressed as linear combinations of the η_1^{o} and η_2^{o} terms and gradients of functions (see [1] for further details). In the main text, we consider the limit $\eta_1^{\mathrm{o}} = -2\eta_2^{\mathrm{o}}$, where we define $\eta_{\mathrm{odd}} \equiv \eta_2^{\mathrm{o}}$. In this case, the force due to the odd viscosities is $\eta_{\mathrm{odd}}(\boldsymbol{e}_z \times \Delta \boldsymbol{v} - \nabla \omega_z)$, where the second (gradient) term can be absorbed into the pressure. In wavenumber space, the odd viscosity term in this limit reads $\eta_{\mathrm{odd}} k^2 \boldsymbol{v}(\boldsymbol{k}) \times \boldsymbol{e}_z$ and can be thought of as a wavenumber-dependent rotation.

Parameters of the direct numerical simulations

Supplementary Table I. Parameters that are used for the simulations in this work for the forward cascade runs (I) and inverse cascade runs (II). Listed are the average energy injection rate $\langle \epsilon \rangle$, normal shear viscosity ν, odd viscosity ν_{odd}, injection wavenumber k_{in}, odd viscosity wavenumber $k_{\mathrm{odd}} = \epsilon^{1/4} \nu_{\mathrm{odd}}^{-3/4}$, grid resolution N^3, Kolmogorov length $\ell_\nu = \epsilon^{-1/4} \nu^{3/4}$, total simulation time T, time-step Δt, Kolmogorov time $\tau_\nu = \epsilon^{-1/2} \nu^{1/2}$ and the *a posteriori* integral scale Reynolds number without odd viscosity Re.

	$\langle \epsilon \rangle$	ν	ν_{odd}	k_{in}	k_{odd}	N^3	$\ell_\nu/(L/N)$	T	Δt	$\tau_\nu/\Delta t$	Re
I	1.4×10^{-5}	9.4×10^{-6}	$[0.3 - 2.4] \times 10^{-3}$	3	$[27 - 6]$	768^3	0.34	2×10^3	$[1.0 - 0.2] \times 10^{-2}$	$[82 - 410]$	1.3×10^3
II	2.1×10^{-6}	9.4×10^{-6}	$[0.5 - 5.0] \times 10^{-3}$	24	$[11 - 2]$	512^3	0.37	8×10^3	$[1.0 - 0.5] \times 10^{-2}$	$[210 - 420]$	6.2×10^1

Modified Taylor-Proudman theorem

The Taylor-Proudman theorem applied to a fluid under rotation implies $\partial_z \boldsymbol{v} = 0$ [2, 3]. In the main text, we have used an extension of this result to the case of a fluid with non-dissipative viscosities. Here, we give the detailed proof of this result for all non-dissipative viscosities compatible with cylindrical symmetry.

We first rewrite the odd force terms in the Navier-Stokes equation Eq. (S.1) in terms of a cross product with $\boldsymbol{e}_z$:

$$\eta_1^{\mathrm{o}} \begin{bmatrix} (\partial_x^2 + \partial_y^2) v_y \\ -(\partial_x^2 + \partial_y^2) v_x \\ 0 \end{bmatrix} = -\eta_1^{\mathrm{o}} \boldsymbol{e}_z \times \Delta_\perp \boldsymbol{v}, \tag{S.2}$$

$$\eta_2^{\mathrm{o}} \begin{bmatrix} -\partial_z^2 v_y - \partial_y \partial_z v_z \\ \\ \partial_z^2 v_x + \partial_x \partial_z v_z \\ \\ \partial_z(\partial_y v_x - \partial_x v_y) \end{bmatrix} + \nabla \omega_z = \eta_2^{\mathrm{o}} \boldsymbol{e}_z \times (\Delta \boldsymbol{v} - 2\Delta_\perp \boldsymbol{v}), \tag{S.3}$$

where $\Delta_\perp \equiv \partial_x^2 + \partial_y^2$. Note that we can add gradient terms to these expressions without modifying the flow. Assuming that the dominant balance is between the odd viscosity term and the pressure gradient term, we write for η_1^{o}:

$$-\eta_1^{\mathrm{o}} \boldsymbol{e}_z \times \Delta_\perp \boldsymbol{v} = \nabla P. \tag{S.4}$$

Next, we take the curl of this equation to remove the pressure term, and simplify the resulting expression by applying the vector calculus identity

$$\boldsymbol{\nabla} \times (\boldsymbol{A} \times \boldsymbol{B}) = \boldsymbol{A}(\boldsymbol{\nabla} \cdot \boldsymbol{B}) - (\boldsymbol{A} \cdot \boldsymbol{\nabla})\boldsymbol{B} + (\boldsymbol{B} \cdot \boldsymbol{\nabla})\boldsymbol{A} - \boldsymbol{B}(\boldsymbol{\nabla} \cdot \boldsymbol{A}).$$

Enforcing incompressibility ($\nabla \cdot \boldsymbol{v} = 0$), we obtain

$$\eta_1^{\mathrm{o}} (\boldsymbol{e}_z \cdot \nabla) \Delta_\perp \boldsymbol{v} = 0, \tag{S.5}$$

which can be simplified into a modified version of the Taylor-Proudman theorem:

$$\partial_z \Delta_\perp \boldsymbol{v} = 0. \tag{S.6}$$

Repeating for η_2^{o}, we obtain

$$\partial_z (\Delta - 2\Delta_\perp) \boldsymbol{v} = 0. \tag{S.7}$$

In the main text, we consider the simplifying limit $\eta_1^{\mathrm{o}} = -2\eta_2^{\mathrm{o}}$, in which case the theorem takes the form

$$\partial_z \Delta \boldsymbol{v} = 0. \tag{S.8}$$

In an incompressible flow, all the odd viscosity coefficients compatible with cylindrical symmetry can be expressed as combinations of η_1^{o} and η_2^{o} (plus gradient terms that can be absorbed into the pressure). As the (modified) Taylor-Proudman theorem relies on the linearized Navier-Stokes equation (i.e., the Stokes equation), we can take linear combinations of Eqs. (S.6)-(S.7) above. Therefore, modified versions of the Taylor-Proudman theorem hold for all odd viscosities compatible with cylindrical symmetry.

Linear stability of the fluid and odd waves

We start with the Navier-Stokes equation

$$\rho D_t v_i = -\partial_i P + \eta_{ijk\ell} \partial_j \partial_\ell v_k \tag{S.9}$$

with the incompressibility condition $\partial_i v_i = 0$, in which $\boldsymbol{v}(t, \boldsymbol{x})$ is the velocity field, ρ is the density, P is the pressure, $\eta_{ijk\ell}$ is the viscosity tensor, $D_t = (\partial_t + v_k \partial_k)$ is the convective derivative, $\partial_k = \partial/\partial x_k$.

To analyze the linear stability of a fluid with odd viscosity, we linearize the Navier-Stokes equations about $\rho = \rho_0$ and $\boldsymbol{v} = \boldsymbol{0}$. We end up with the incompressible Stokes equations

$$\rho_0 \partial_t v_i = -\partial_i P + \eta_{ijk\ell} \partial_j \partial_\ell v_k \tag{S.10}$$

$$\partial_i v_i = 0. \tag{S.11}$$

Considering solutions of the form $\mathrm{e}^{st + \mathrm{i}\boldsymbol{k} \cdot \boldsymbol{x}}$, where $s = \sigma + \mathrm{i}\omega$ is a complex growth rate, we rewrite them in Fourier space:

$$s \rho_0 v_i = -\mathrm{i} k_i P - \eta_{ijk\ell} k_j k_\ell v_k \tag{S.12}$$

$$k_i v_i = 0. \tag{S.13}$$

Using the incompressibility condition, we solve for the pressure by multiplying by k_i, and find

$$P = \mathrm{i} \eta_{ijk\ell} \frac{k_i k_j k_\ell}{k^2} v_k. \tag{S.14}$$

Plugging this back into the Stokes equation, we arrive at an equation just for the velocity:

$$\rho_0 s v_n = \eta_{ijk\ell}\frac{k_n k_i k_j k_\ell}{k^2} v_k - \eta_{njk\ell} k_j k_\ell v_k \equiv M_{nk}(\boldsymbol{k}) v_k. \tag{S.15}$$

The dispersion relation $s(\boldsymbol{k}) = \sigma(\boldsymbol{k}) + \mathrm{i}\omega(\boldsymbol{k})$ is then given by the eigenvalues of $M_{nk}(\boldsymbol{k})/\rho_0$.

In the case of Eq. (S.1) where the two independent odd viscosities η_1^{o} and η_2^{o} are present in addition to the normal shear viscosity η, we obtain

$$\rho_0 \omega(\boldsymbol{k}) = \pm \frac{\eta_1^{\mathrm{o}} k_z (k_x^2 + k_y^2) + \eta_2^{\mathrm{o}} k_z (k_x^2 + k_y^2 - k_z^2)}{|k|} \tag{S.16}$$

$$\rho_0 \sigma(\boldsymbol{k}) = -\eta k^2. \tag{S.17}$$

In the main text, we consider the case $\eta_1^{\mathrm{o}} = -2\eta_2^{\mathrm{o}}$, in which case the above dispersion relations reduce to the dispersion given in the main text, with $\nu_{\mathrm{odd}} \equiv \eta_2^{\mathrm{o}}/\rho_0$.

Helical decomposition

In this section, we describe the decomposition of the fluxes into different manifolds according to their helical signature used in the main text. Following [4], we decompose the total net flux $\Pi(k) = \Pi_{\mathrm{homo}}(k) + \Pi_{\mathrm{hete}}(k)$ into a component due to triads with the same sign of helicity Π_{homo} and triads with opposing signs of helicity Π_{hete}. To that extent, we decompose the flow into positively and negatively helical modes (as is also laid out in the section *Effect of odd waves on the non-linear energy transfer*)

$$\begin{aligned} \boldsymbol{v}(\boldsymbol{k},t) &= \boldsymbol{v}_+(\boldsymbol{k},t) + \boldsymbol{v}_-(\boldsymbol{k},t) \\ &= v_+(\boldsymbol{k},t)\boldsymbol{h}^+(\boldsymbol{k}) + v_-(\boldsymbol{k},t)\boldsymbol{h}^-(\boldsymbol{k}), \end{aligned} \tag{S.18}$$

Then the homochiral energy flux is obtained as

$$\Pi_{\mathrm{homo}}(k) \equiv \Pi_{(+,+,+)}(k) + \Pi_{(-,-,-)}(k), \tag{S.19}$$

with

$$\begin{aligned} &\Pi_{(\pm,\pm,\pm)}(k) \\ &= \mathrm{Im} \sum_{|\boldsymbol{k}|<k} \sum_{\boldsymbol{p}+\boldsymbol{q}=\boldsymbol{k}} v_{i,\pm}^*(\boldsymbol{k},t) P_{ij}(\boldsymbol{k}) q_\ell v_{\ell,\pm}(\boldsymbol{p},t) v_{j,\pm}(\boldsymbol{q},t), \end{aligned} \tag{S.20}$$

and the other contributions to total flux are from heterochiral triads

$$\Pi_{\mathrm{hete}}(k) = \Pi(k) - \Pi_{\mathrm{homo}}(k). \tag{S.21}$$

It is known that in 3D turbulence, the heterochiral triads that dominate the energy flux primarily cascade energy directly, while the typically subleading homochiral triads primarily cascade energy inversely [4, 5].

Since we have seen that odd viscosity can manipulate the direction of the turbulent cascade, the question naturally arises how the odd viscosity modifies the relative importance of both helical manifolds. For the case of the direct cascade, we see in ED Fig. 1a(i,ii) that as the cascade approaches k_{odd}, the homochiral flux becomes stronger, in agreement with the notion that the flow develops a tendency to cascade energy inversely. Consequentially, as the energy starts to pile up, also the heterochiral flux is enhanced. This once more showcases the competition between the inverse cascading tendency and direct cascade that leads to the observed energetic condensation. For the inverse cascading cases in ED Fig. 1a(iii,iv), this becomes even more striking. There, we observe that the odd viscosity produces an inverse cascade in the homochiral manifold, which is in turn balanced by a forward cascade in the heterochiral manifold, such that the total net flux vanishes in the inverse range. This self-regulating mechanism is referred to as a flux loop state [6], where kinetic energy is consistently looping into the condensate through the homochiral flux and then back again through the heterochiral flux. Note that to evidence this, we have employed hyperdissipation to mimic more scale separation between the dissipative scales and injection scales.

This marks an important difference between the odd viscosity condensates resulting from a direct cascade and those resulting from an inverse cascade: while the former are saturated by dissipation as treated in the main text, the condensates from the inverse cascade are saturated by the flux balance between the helical manifolds. However, the significant magnitude of the flux in both helical manifolds indicates that also the condensates in the inverse cascading case are strongly out-of-equilibrium states.

Statistical steady-state of the cascade

To confirm that we have obtained a statistical steady-state in our simulations, we assess the transient energetic content of the first modes, which are the slowest modes to equilibrate, shown in Supplementary Fig. 1. This confirms that the dynamics in the averaging window is indeed governed by fluctuations around a statistically steady state.

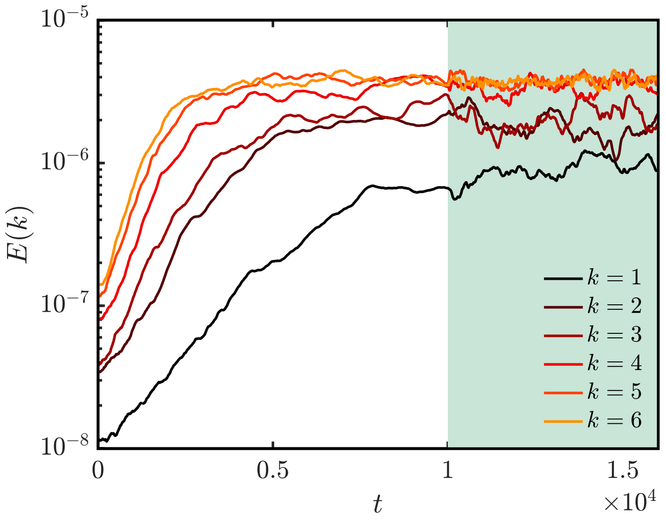

Supplementary Figure 1. Transient energetic content of the first six modes for the inverse cascade case with $\nu_{\mathrm{odd}}/\nu = 212$. The green highlighted interval denotes the averaging window over which the spectra are obtained.

Odd hyperviscosity

The crucial ingredient to observe the described turbulent pattern formation is the competing direct cascade (at small k) and inverse cascade (at large k), and a non-dissipative mechanism that discriminates between the two. In that regard, the scaling theories presented in this work can be straightforwardly extended to odd hyperviscosities of the form $\tilde{\eta}_{\mathrm{odd}}\boldsymbol{e}_z \times \Delta^p \boldsymbol{v}$ with power $p > 1$, which would make the transition from 3D to quasi-2D arbitrarily sharp/smooth, rendering also the region of spectral condensation arbitrarily peaked. This provides an ideal model system to study more complex natural flow systems in which a variety of different multi-scale mechanisms could exists that produce competing cascades, with varying sharp or smooth transitions between them that are different from the $\sim k^2$ scaling.

For arbitrary p, we find for the dispersion of odd waves

$$\tilde{\omega}(\boldsymbol{k}) = \pm \tilde{\nu}_{\mathrm{odd}} k_z |k|^{2p-1} \,, \tag{S.22}$$

yielding for the odd viscosity scale, where condensation begins

$$\tilde{k}_{\mathrm{odd}} = \epsilon^{1/(6p-2)} \tilde{\nu}_{\mathrm{odd}}^{-3/(6p-2)} , \tag{S.23}$$

and for the condensation spectrum

$$\tilde{E}(k) \sim \epsilon^{1/2} \tilde{\nu}_{\mathrm{odd}}^{1/2} \, k^{p-2} \qquad (k \gg \tilde{k}_{\mathrm{odd}}), \tag{S.24}$$

until the condensation peak is attained at

$$\tilde{k}_c \sim \left(\epsilon^{1/2} \nu^{-1} \tilde{\nu}_{\mathrm{odd}}^{-1/2} \right)^{1/(p+1)} . \tag{S.25}$$

While the main text treats the case $p = 1$ where the transition from a direct cascade at small k to an inverse cascade at large k is smooth, we can also consider the other extreme, where the transition is fully sharp (semantically, the limit $p \to \infty$). Here the odd viscosity term $\eta_{\mathrm{odd}} \boldsymbol{e}_z \times k^2 \boldsymbol{v}$ in the Navier-Stokes equations is replaced by $\tilde{\eta}_{\mathrm{odd}} \theta(|\boldsymbol{k}| - |\tilde{\boldsymbol{k}}_{\mathrm{odd}}|) \boldsymbol{e}_z \times \boldsymbol{v}$ where $\tilde{k}_{\mathrm{odd}}$ is a set wavenumber in the inertial range and $\theta(|\boldsymbol{k}| - |\tilde{\boldsymbol{k}}_{\mathrm{odd}}|)$ is a step function which is non-zero for $|\boldsymbol{k}| > |\tilde{\boldsymbol{k}}_{\mathrm{odd}}|$. Since the transition is now sharp, the start of condensation and peak condensation wavenumbers coincide $\tilde{k}_c = \tilde{k}_{\mathrm{odd}}$, leading to maximum condensation sharply at a single wavelength. Numerical results for this case are provided in ED Fig. 1e, which indeed shows a sharp condensation spectrum around $\tilde{k}_c$, as well as a diffusive equipartitioned scaling $\sim k^2$ to the left of the condensation peak, akin to what is observed in 3D turbulence on wavenumbers smaller than the injection scale. As a result, the associated pattern in the vorticity field in ED Fig. 1f is more isotropic than in Fig. 3b, which is consistent with the prediction of aspect ratio $\gamma = 1$ from Eq. (26) in the Methods.

Rossby wave turbulence

In this section, we review the system of 2D turbulence under the influence of Rossby waves and point out the similarities and differences with the case of odd fluids.

We consider an incompressible fluid with Cartesian coordinates $(x,\, y)$. In the so-called β-plane approximation, the Coriolis force depends linearly on the coordinate y with a coefficient β. Namely, it is $\boldsymbol{f}_{\text{c}} = -\beta\, y\, \epsilon \cdot \boldsymbol{v}$. The Navier-Stokes equation reads

$$\partial_t \boldsymbol{v} + (\boldsymbol{v}\cdot\boldsymbol{\nabla})\boldsymbol{v} - \beta\, y\, \epsilon\cdot\boldsymbol{v} + \boldsymbol{\nabla} p = -\alpha\boldsymbol{v} + \nu\Delta\boldsymbol{v} + \boldsymbol{f} \tag{S.26}$$

in which $\boldsymbol{v}$ is the velocity field, p the pressure field, ϵ the Levi-Civita symbol, ν the kinematic viscosity (a diffusion coefficient), α a friction term, and $\boldsymbol{f}$ a body force representing the velocity forcing.

Taking the curl of the Navier-Stokes equation, we find

$$\partial_t\omega + [\epsilon_{ij}(\partial_i v_k)(\partial_k v_j) + v_k\partial_k\omega] + \beta v_i(\partial_i y) = -\alpha\omega + \nu\Delta\omega + f_\omega \tag{S.27}$$

where $\omega = \nabla\times v = \epsilon_{ij}\partial_i v_j = \partial_x v_y - \partial_y v_x$ is the 2D vorticity, and $f_\omega = \epsilon_{ij}\partial_i f_j$ is a vorticity forcing. The term $\epsilon_{ij}(\partial_i v_k)(\partial_k v_j)$ vanishes because of incompressibility, so we end up with

$$\partial_t\omega + (\boldsymbol{v}\cdot\nabla)\omega + \beta v_y = -\alpha\omega + \nu\Delta\omega + f_\omega \tag{S.28}$$

in which we have used $\boldsymbol{v}\cdot(\boldsymbol{\nabla} y) = v_y$.

As we have an incompressible 2D flow, it is convenient to introduce a streamfunction ψ such that

$$\boldsymbol{v} = \begin{pmatrix} v_x \\ v_y \end{pmatrix} = \begin{pmatrix} -\partial_y\psi \\ \partial_x\psi \end{pmatrix} = -\epsilon\cdot\boldsymbol{\nabla}\psi \tag{S.29}$$

In addition, the 2D vorticity is $\omega = \Delta\psi$.

We introduce the Jacobian (Poisson bracket)

$$J(a,b) = \frac{\partial a}{\partial x}\frac{\partial b}{\partial y} - \frac{\partial a}{\partial y}\frac{\partial b}{\partial x}. \tag{S.30}$$

It allows us to express the convective term in terms of the streamfunction ψ as

$$J(\psi, b) = (\boldsymbol{v}\cdot\nabla)b \tag{S.31}$$

Then, the Navier-Stokes equation becomes

$$\partial_t\omega + J(\psi,\omega) + \beta\partial_x\psi = -\alpha\omega + \nu\Delta\omega + f_\omega. \tag{S.32}$$

As $\omega = \Delta\psi$, this equation is entirely written in terms of the stream function. Equation (S.32) is known as the Charney-Hasegawa-Mima (CHM) equation [7–14].

Removing the driving and dissipative terms, we get

$$\partial_t\omega + J(\psi,\omega) = -\beta\partial_x\psi \tag{S.33}$$

with $\omega = \Delta\psi$. Let us first consider the linearized equation

$$\partial_t\omega = -\beta\partial_x\psi. \tag{S.34}$$

It has wave solutions called Rossby waves or drift waves, with dispersion relation

$$\Omega(\mathbf{k}) = -\beta\frac{k_x}{k^2}. \tag{S.35}$$

Now, we consider the decomposition of the streamfunction into the corresponding modes

$$\psi(t,x) = \sum_{\boldsymbol{k}} \psi(t,\boldsymbol{k})\, e^{i[\Omega(\boldsymbol{k})t - \boldsymbol{k}\cdot\boldsymbol{r}]} \tag{S.36}$$

with a reality condition so that this is real (this is the equivalent of the decomposition in helical waves used in the Methods section *Effect of odd waves on the non-linear energy transfer*).

We get

$$\partial_t\psi_{\mathbf{k}} = \sum_{\mathbf{k}+\mathbf{p}+\mathbf{q}=\mathbf{0}} C_{k|p,q}\, e^{-i[\Omega(p)+\Omega(q)+\Omega(k)]t}\psi^*_{\mathbf{p}}\psi^*_{\mathbf{q}} \tag{S.37}$$

in which

$$C_{k|p,q} = \frac{1}{2}\frac{(p_x q_y - p_y q_x)(q^2-p^2)}{k^2} \tag{S.38}$$

A characteristic advective timescale is $\tau^{-1}(k) = k v_k \sim kU$ where U is a characteristic velocity. Comparing with the Rossby wave frequency $\Omega(\boldsymbol{k})$ with $k_x \sim k$, we find a critical wavenumber k_{R} such that $\Omega \sim \tau^{-1}$ leading to $k_{\mathrm{R}} \equiv \sqrt{\beta/U}$ (called Rhines wavenumber). For wavenumbers $k \lesssim k_{\mathrm{R}}$, the non-linear energy transfer is reduced because of the decorrelation of the triads by Rossby waves, except for modes with $\Omega = 0$. This leads to a one-dimensionalization of the flow, and to the appearance of a scale $\sim 1/k_{\mathrm{R}}$ [15–22]. This is illustrated in ED Fig. 2, in which we simulate Eq. (S.32) using the open-source pseudospectral solver Dedalus [23].

Exact solution of the equilibrium mass transfer model

In this section, we determine an exact solution of mass transfer model with no flux ($J_{\mathrm{in}} = J_{\mathrm{out}} = 0$). Setting $J_n \equiv J^{+,n} - J^{-,n} = 0$ yields the recurrence relation

$$c_{n+1} = k^{+,n}/k^{-,n} c_n^2. \tag{S.39}$$

Defining $\alpha_n \equiv k^{+,n}/k^{-,n}$ and iterating, we find that

$$c_{n+1} = \left[\prod_{k=0}^{n-1} \alpha_{n-k}^{2^k}\right] c_1^{2^n}. \tag{S.40}$$

With our choice Eq. (34), we find

$$\alpha_n \equiv \frac{k^{+,n}}{k^{-,n}} = \frac{\kappa_n^+/2}{\kappa_{n+1}^-} = \frac{1}{2}\frac{\kappa_0^+ + \kappa_1^+ \frac{N-n}{N-1}}{\kappa_0^- + \kappa_1^- \frac{n}{N-1}} \tag{S.41}$$

We then use the identity

$$\prod_{k=0}^{n-1} (a+k)^{2^k} = a e^{2^n \Phi^{(0,1,0)}(2,0,a+n) - 2\Phi^{(0,1,0)}(2,0,a+1)} \tag{S.42}$$

in which $\Phi(z,s,a)$ is Lerch's Transcendent [24, §25.14] and $\Phi^{(0,1,0)}$ its first derivative with respect to the second argument to find

$$\begin{aligned} c_{n+1} = -\frac{a_n^+}{a_n^-} c_1^{2^n} \left(\frac{\kappa_1^+}{2\kappa_1^-}\right)^{2^n-1} \times \\ \exp\Bigg[2^n[\Phi^{(0,1,0)}(2,0,n+a_+) - \Phi^{(0,1,0)}(2,0,n+a_-)] \\ + 2[\Phi^{(0,1,0)}(2,0,1+a_-) - \Phi^{(0,1,0)}(2,0,1+a_+)]\Bigg] \end{aligned} \tag{S.43}$$

in which $a_n^+ = (N-1)(\kappa_0^+/\kappa_1^+) - n + N$ and $a_n^- = -(N-1)(\kappa_0^-/\kappa_1^-) - n$. (Numerically, iterating the recurrence relation (S.39) is more convenient than evaluating directly Eq. (S.40) or Eq. (S.43), as doing so requires some care to avoid floating point issues.)

[1] T. Khain, C. Scheibner, M. Fruchart, and V. Vitelli, Journal of Fluid Mechanics **934**, A23 (2022).
[2] G. I. Taylor, Proceedings of the Royal Society of London **104**, 213 (1923).
[3] J. Proudman, Proceedings of the Royal Society of London **92**, 408 (1916).
[4] F. Waleffe, Physics of Fluids A: Fluid Dynamics **4**, 350–363 (1992).
[5] L. Biferale, S. Musacchio, and F. Toschi, Physical Review Letters **108**, 164501 (2012).
[6] A. Alexakis and L. Biferale, Physics Reports **767**, 1 (2018).
[7] C. Connaughton, S. Nazarenko, and B. Quinn, Physics Reports **604**, 1–71 (2015).
[8] G. Boffetta, F. D. Lillo, and S. Musacchio, Europhysics Letters (EPL) **59**, 687–693 (2002).
[9] E. Tassi, C. Chandre, and P. J. Morrison, Physics of Plasmas **16**, 10.1063/1.3194275 (2009).

[10] A. Hasegawa and K. Mima, Physical Review Letters **39**, 205–208 (1977).
[11] J. G. Charney, Journal of the Atmospheric Sciences **28**, 1087–1095 (1971).
[12] W. Horton, Reviews of Modern Physics **71**, 735–778 (1999).
[13] J. Pedlosky 10.1007/978-1-4684-0071-7 (1979).
[14] B. Galperin, S. Sukoriansky, and N. Dikovskaya, Physica Scripta **T132**, 014034 (2008).
[15] P. H. Diamond, S.-I. Itoh, K. Itoh, and T. S. Hahm, Plasma Physics and Controlled Fusion **47**, R35 (2005).
[16] S. Sukoriansky, N. Dikovskaya, and B. Galperin, Journal of the Atmospheric Sciences **64**, 3312 (2007).
[17] P. Berloff, I. Kamenkovich, and J. Pedlosky, Journal of Fluid Mechanics **628**, 395 (2009).
[18] A. Chekhlov, S. A. Orszag, S. Sukoriansky, B. Galperin, and I. Staroselsky, Physica D: Nonlinear Phenomena **98**, 321 (1996).
[19] P. B. Rhines, Journal of Fluid Mechanics **69**, 417 (1975).
[20] P. B. Rhines, Annual Review of Fluid Mechanics **11**, 401 (1979).
[21] B. Legras, B. Villone, and U. Frisch, Physical Review Letters **82**, 4440 (1999).
[22] N. Grianik, I. M. Held, K. S. Smith, and G. K. Vallis, Physics of Fluids **16**, 73 (2004).
[23] K. J. Burns, G. M. Vasil, J. S. Oishi, D. Lecoanet, and B. P. Brown, Physical Review Research **2**, 023068 (2020).
[24] DLMF, *NIST Digital Library of Mathematical Functions*, `https://dlmf.nist.gov/`, Release 1.1.10 of 2023-06-15 (2023), f. W. J. Olver, A. B. Olde Daalhuis, D. W. Lozier, B. I. Schneider, R. F. Boisvert, C. W. Clark, B. R. Miller, B. V. Saunders, H. S. Cohl, and M. A. McClain, eds.
